# Synthesis of Fe_3_O_4_@SiO_2_@Pr-NH_2_@DAP as a magnetic recyclable nano-catalyst for efficient synthesis of pyranothiazolopyrimidines and 4*H*-pyrans under solvent-free condition

**DOI:** 10.1038/s41598-023-41793-z

**Published:** 2023-09-11

**Authors:** Maryam Danehchin, Abbas Ali Esmaeili

**Affiliations:** Department of Chemistry, Faculty of Science, Ferdowsi University of Mashhad, P.O. Box 9177948974, Mashhad, Iran

**Keywords:** Chemistry, Nanoscience and technology

## Abstract

In this research, we describe the synthesis of silica-coated nano-Fe_3_O_4_ particles, which were successfully modified by diaminopyrimidine, and their physicochemical properties were characterized using FT-IR, XRD, TEM, FE-SEM, EDX, EDX-mapping, and TGA. The catalytic activity of this novel nano-catalyst was evaluated by three-component reactions for the preparation of pyranothiazolopyrimidines and 4*H*-pyrans under solvent-free conditions. Recyclability of the catalyst up to six consecutive rounds, atom economy, high yield and purity of desired products, and easy work-up method are some of the exciting features of this system that make it more favorable from a green chemistry point of view.

## Introduction

Over the last few decades, the application of heterogeneous catalysts in various organic reactions has been extensively studied because of their industrial importance and environmentally friendly features. On the other hand, heterogeneous catalysts can be recovered and separated from the product. Since heterogeneous catalysts are not in the same phase with reactants as homogeneous catalysts, their catalytic performance is commonly reduced over time (the total reaction efficiency is reduced)^1–6^.

Therefore, the construction of heterogeneous catalysts from hybrids organic–inorganic is of great interest due to their high structural diversity, flexibility, and creation of high mechanical and thermal stability^7–10^.

Recent research has shown that decreasing heterogeneous catalyst particles to nano-size (1–100 nm) can improve the catalyst's quality and increase the active surface area, leading to increased reactivity of catalysts^11–13^. From green chemistry, developing new catalyst recycling methods to replace conventional approaches such as centrifugation and filtration methods is useful. Therefore, to solve this problem, magnetic nanocatalysts became the strong candidate due to their unique rapid separation from the liquid medium by an external magnet^14–20^. Magnetic Fe_3_O_4_ nanocrystals (NCs) have other unique advantages such as low toxicity, eco-friendly nature, reusability, powerful chemical and thermal tension stability, and high surface area; mostly scalable and cost-effective. They are readily prepared by the co-precipitation technique; therefore, it is the most suitable candidate for catalyst support^21–23^. Today, silicon dioxide (SiO_2_), functionalized with diverse linkers or coupling agents, is one of the best materials widely used for the shell of Fe_3_O_4_ NPs, owing to its excellent stability biocompatibility, and improved reactivity. The 2,4-diamino-pyrimidine scaffold that bonded to the catalyst is a pyrimidine derivative that contains two amino groups, which accelerate the basicity of the catalyst. For this purpose and to increase the reactivity, a linker should be used to bind the amine compound to the silica shell^24,25^.

Multicomponent reactions (MCRs) are considered an effective and powerful tool for synthesizing new heterocyclic compounds using a simple process. Another advantage of these reactions is that they are atomically efficient and are usually performed under mild conditions^26–28^. Complex molecules such as heterocyclic compounds offer fast and experimentally simple methods and are also easily assessable using the MCRs.

Among many biologically active heterocyclic compounds, 4*H*-pyran derivatives constitute a significant group of organic compounds due to their possible biological activities^29,30^. These heterocyclic compounds have access to a wide variety of activities such as anticoagulant, spasmolytic, diuretic, spasmolytic, anticancer, antipyretic, anti-hyperglycemic, and anti-dyslipidemic activities^31^. In addition, fused 4*H*-pyran derivatives such as pyranopyrimidine represent a wide range of applications in the pharmaceutical industry, and biological activities such as antitumor, anticancer, antifungal, antioxidant, and antihypertensive^32–35^.

Besides, thiazolopyrimidines have been considered a valuable scaffold with various biological activities such as antibacterial, anticancer, antidiabetic, *anti*-HSV-1, antibacterial, antimicrobial, antioxidant, antimalarial, *anti*-HIV, herbicidal, and antiviral agents^36–38^. Several methods have been used for the synthesis of 4*H*-pyran derivatives including the reaction between 1,3-dicarbonyl compounds, different aldehydes, and malononitrile under different conditions^39–42^. Low yield, costly catalysts, toxic solvent, tedious work-up, high reaction times, and complicated catalyst recovery are drawbacks of these procedures.

**Figure 1:**
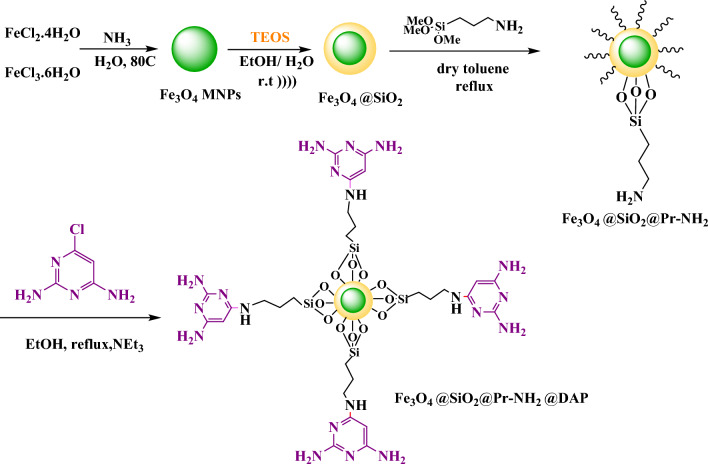
Total procedure for the synthesis of Fe_3_O_4_@SiO_2_@Pr-NH_2_@DAP.

**Figure 2:**
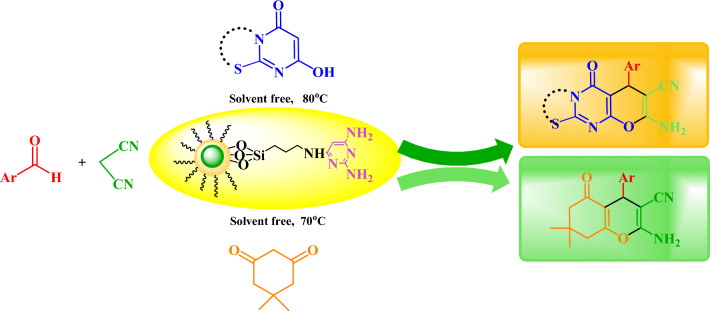
Synthesis of 4*H*-pyran derivatives using novel Fe_3_O_4_@SiO_2_@Pr-NH_2_@DAP as a catalyst.

In continuation to our endeavor to develop a green synthetic protocol and assess the heterocyclic compounds^43–47^, we introduced Fe_3_O_4_@SiO_2_-functionalized with diaminopyrimidine (DAP) as a novel and inexpensive heterogeneous magnetic nano-catalyst (Fig. 1) for facile and rapid synthesis of efficient synthesis of pyranothiazolopyrimidines and 4*H*-pyran derivatives (Fig. 2).

## Experimental section

### Materials and apparatus

All chemicals were purchased from commercial sources and used without purification. Melting points were determined using an Electrothermal 9100 instrument. Infrared (IR) spectra were acquired on a Nicolet Avatar 370 FT-IR Therma spectrometer in cm^−1^ with spectroscopic grade KBr. The ^1^HNMR and ^13^C NMR (300 MHz and 75 MHz, respectively) were obtained on a Bruker Avance DPX-300 instrument. MS was done using Varian Mat CH-7 at 70 eV. Transmission Electron Microscope (TEM) with EM10C (100 kV) microscope (Zeiss, Germany) was used for characterizing the size morphology of nanoparticles. FE-SEM images, EDS, and EDS-mapping were recorded on a Leo 1450 VP scanning electron microscope equipped with an SC7620 energy-dispersive spectrometer (SEM-EDS) presenting a 133-eV resolution at 20 kV. The crystal structure of the products was characterized by X-ray diffraction (XRD) D8 ADVANCE Bruker diffractometer with monochromated at 40 kV and 30 mA, using CuKα radiation (k = 0.154 Å), in the 2θ range from 10° to 80°. Thermogravimetric analysis (TGA) experiments were carried out on a Shimadzu Thermogravimetric Analyzer (TG-50) in the temperature range from room temperature to 600 °C under a nitrogen atmosphere at 10 °C/min heating rate. To measure the magnetic feature of the catalyst, we utilized a vibrating sample magnetometer (VSM; model 7400, Lake Shore).

## Procedures

### Preparation of Fe_3_O_4_ nanoparticles

Fe_3_O_4_ nanoparticles (NPs) were synthesized by a co-precipitation method which can be summarized as follows: 1.0 g of Iron(II) chloride tetrahydrate (FeCl_2_·4H_2_O) was added to a stirred mixture of Iron(III) chloride hexahydrate (FeCl_3_·6H_2_O, 2.5 g) in deionized water (70 mL) in ambient temperature. The mixture was slowly heated to 60 °C, and then ammonia (20 mL) was added dropwise to the resulting compound with vigorous stirring under the nitrogen atmosphere. The resulting black precipitate was stirred continuously for 60 min, and then the generated precipitate was separated and washed several times with deionized water and ethanol, then dried under vacuum for 24 h.

### Preparation of silica-coated Fe_3_O_4_ magnetic nanoparticles (Fe_3_O_4_@SiO_2_)

At first, 2.0 g of Fe_3_O_4_ nanoparticles were diluted with 120 mL of deionized water and 250 mL of ethanol, and the mixture was dispersed for 30 min. Then 25% ammonium hydroxide solution (10 mL) was added to the reaction mixture at ambient temperature and was stirred vigorously under a nitrogen atmosphere. Subsequently, 2.0 mL TEOS was dropwise added to this dispersion, and it was stirred strongly for 12 h at ambient temperature. The product was separated by an external magnet and washed several times with deionized water and ethanol, subsequently dried under vacuum at 90 °C overnight.

### Preparation of (Fe_3_O_4_@SiO_2_@Pr-NH_2_) nanoparticles

1 mL of 3-aminopropyl trimethoxy silane (APTMS) was added dropwise to 1 g of Fe_3_O_4_@SiO_2_ nanoparticles dispersed in 25 mL of dry toluene with stirring under N_2_ atmosphere. In the next step, the mixture was refluxed at 80 °C for 24 h. Finally, the solid product was collected and washed several times with toluene, and dried in a vacuum oven for 24 h.

### Synthesis of Fe_3_O_4_@SiO_2_@Pr-NH_2_@DAP MNPs

At first, 1 g of Fe_3_O_4_@SiO_2_@Pr-NH_2_ in 50 mL of ethanol was dispersed under ultrasonic conditions for 30 min. Then 5.9 mmol (0.87 g) of 6-chloropyrimidine-2,4-diamine and a catalytic amount of triethylamine were added, and the reaction mixture was stirred under an N_2_ atmosphere for 24 h at 80 °C. Then, the resulting product was collected by an external magnet, washed with ethanol and deionized water, and finally dried for 24 h in a vacuum oven.

### General procedure for the preparation of 4-aryl-2-amino-3-cyano-7,7-dimethyl-5-oxo-5,6,7,8-tetrahydro-4*H*-chromenes (6)

Fe_3_O_4_@SiO_2_@Pr-NH_2_@DAP MNPs (0.04 g) were added to a stirred mixture of aromatic aldehydes (1.0 mmol), Dimedone (1.0 mmol), and malononitrile (1.0 mmol), and heated at 70 °C under solvent-free condition. After the reaction was completed, as monitored by TLC, the reaction mixture was cooled to room temperature, and then hot ethanol (10 mL) was added. Subsequently, the nano-catalyst was separated by an external magnet, washed three times with ethanol, and dried in an oven at 80 °C to be ready for the next run. Then, the filtrate was concentrated, and the resulting residue was purified by crystallization in ethanol to yield **6a**–**k**.

**Figure 3:**
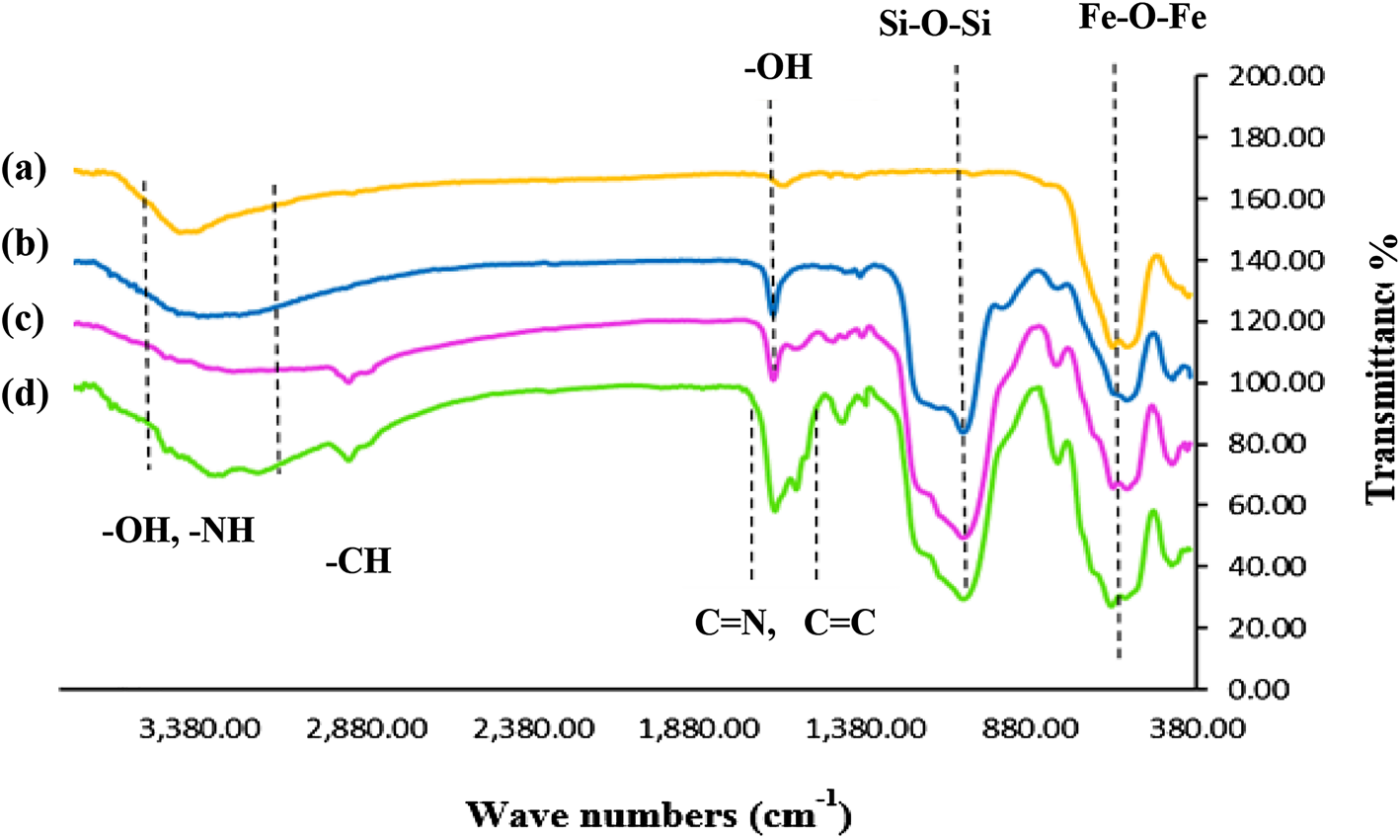
FT-IR spectra: (**a**) Fe_3_O_4_, (**b**) Fe_3_O_4_@SiO_2_, (**c**) Fe_3_O_4_@SiO_2_@Pr-NH_2_, (**d**) Fe_3_O_4_@SiO_2_@Pr-NH_2_@DAP, and *FT-IR* Fourier-transform infrared spectroscopy, *DAP* diaminopyrimidine.

FT-IR spectra of NPs are shown in Fig. 3, to confirm the synthesis of Fe_3_O_4_, Fe_3_O_4_@SiO_2_, Fe_3_O_4_@SiO_2_@-Pr-NH_2,_ and Fe_3_O_4_@SiO_2_@Pr-NH_2_@DAP. FT‐IR spectrum of Fe_3_O_4_ (Fig. 3a) shows a strong band at 583 cm^−1^, which shows the vibration of the Fe–O band, and a peak at 969 cm^−1^ is assigned to bending vibration of the silanol group (Si–OH) while the sharp peak at 1071 cm^−1^ corresponded to the stretching vibration bands of Si–O–Si. The peaks at 1623 cm^−1^ and broad adsorption band at 3440 cm^−1^ are associated with bending (O–H) and stretching (H–O–H) vibration modes of water respectively (Fig. 3b). The spectrum of the Fe_3_O_4_@SiO_2_@Pr–NH_2_ shows peaks at 2880 cm^−1^ and 2932 cm^−1^, due to the C–H stretching vibration of the alkyl chain of amine, while the two broad bands at 1583 and 3423 cm^−1^ are ascribed the N–H bending and stretching vibrations, respectively (Fig. 3c). In the spectra of final product Fe_3_O_4_@SiO_2_@Pr-NH_2_@DAP in Fig. 3d, in addition to the mentioned peaks for Fe_3_O_4_@SiO_2_@Pr-NH_2_, the peaks at 1485 and 1650 cm^−1^ correspond to the C=C and C=N stretching vibrations of DAP, which shows the presence of diaminopyrimidine in the nanocatalyst. The bands at 3334 and 3195 cm^−1^ correspond to the amino groups' asymmetric and symmetric N–H vibrations (Fig. 3d).

### FE-SEM analysis

**Figure 4:**
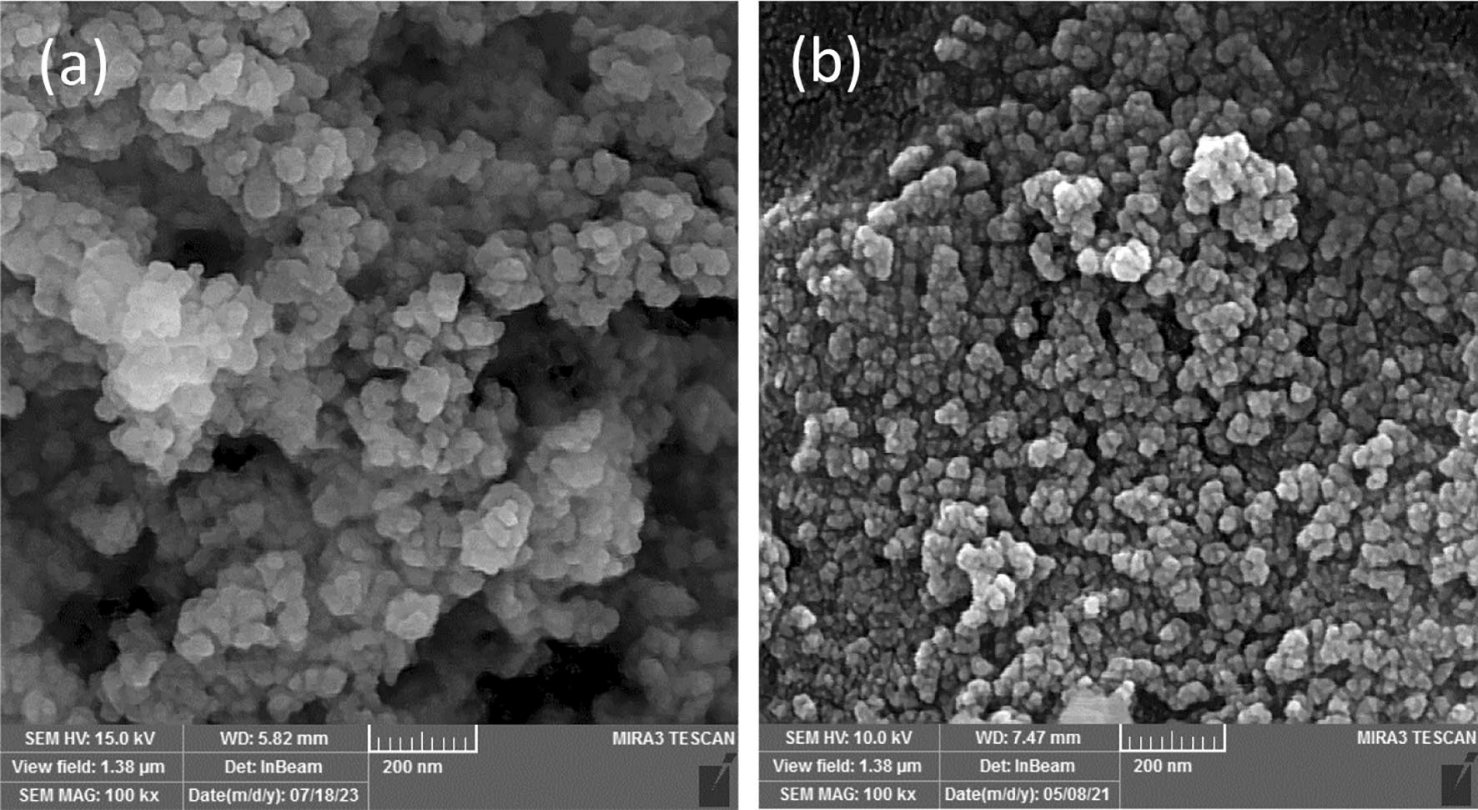
FE-SEM images of (**a**) Fe_3_O_4_@SiO_2_@Pr-NH_2_ and (**b**) Fe_3_O_4_@SiO_2_@Pr-NH_2_@DAP nano-particles.

The surface morphologies of the synthesized materials were examined by field emission scanning electron microscopy (FE-SEM) as shown in Fig. 4a,b. FE-SEM results for Fe_3_O_4_@SiO_2_@Pr-NH_2_, (Fig. 4a) show spherical, narrowly distributed, and well-dispersed aggregated and semi-spherical Fe3O4 particles. Slight changes were observed in the surface morphology for diaminopyrimidine-functionalized magnetic nano silica (Fe_3_O_4_@SiO_2_@Pr-NH_2_@DAP) nanocomposites. After functionalization, there was an observed increase in the surface roughness (Fig. 4b).

**Figure 5:**
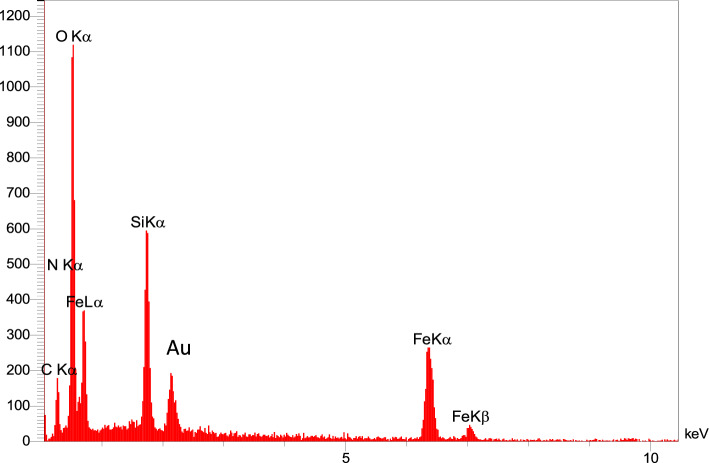
Elemental analysis of Fe_3_O_4_@SiO_2_@Pr-NH_2_@DAP using EDX.

**Figure 6:**
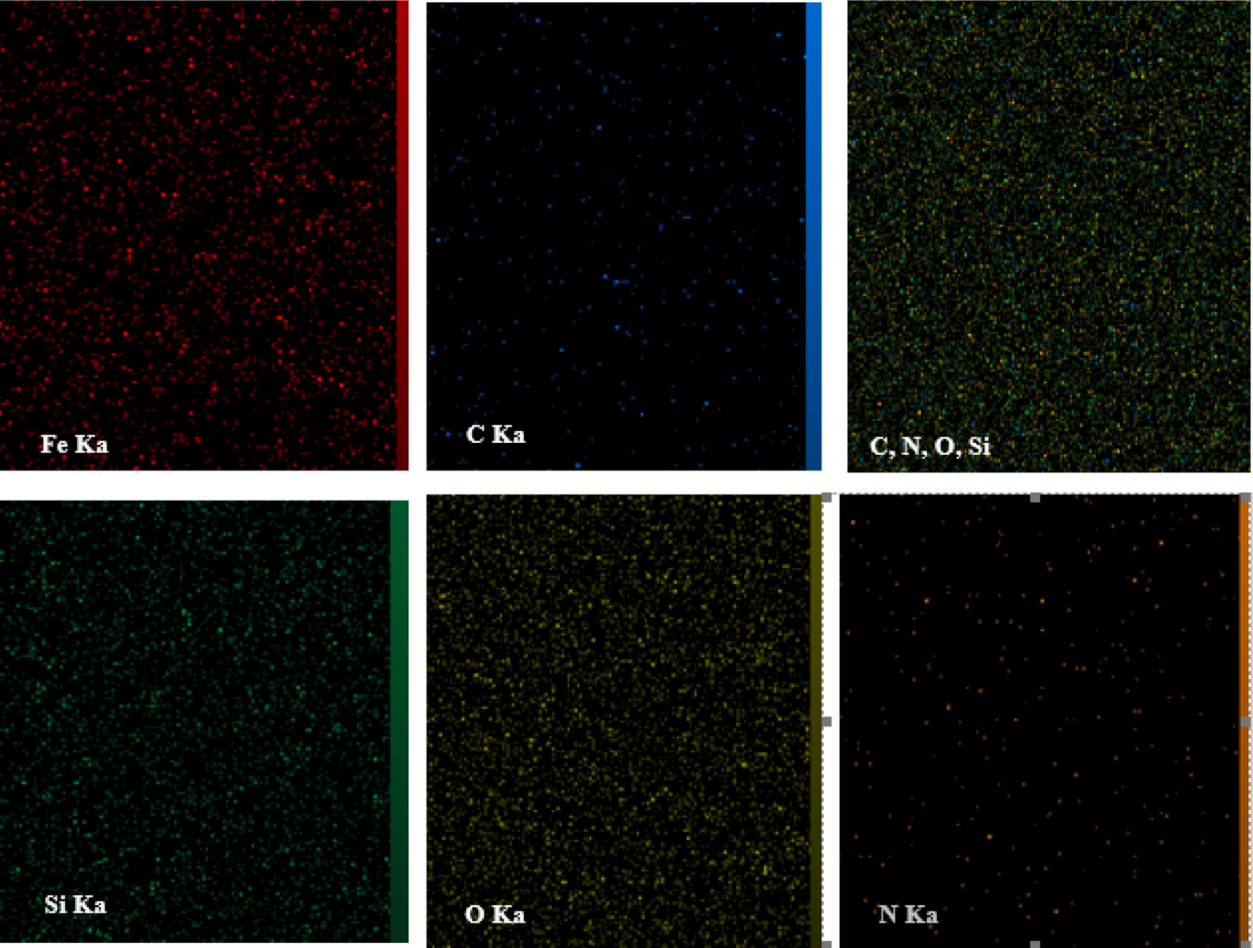
EDX-mapping analysis of Fe_3_O_4_@SiO_2_@Pr-NH_2_@DAP.

To investigate the types of present elements in the structure of Fe_3_O_4_@SiO_2_@Pr-NH_2_@DAP, the energy-dispersive X-ray (EDX) spectrum was recorded and established in Fig. 5. As can be seen, the Fe_3_O_4_@SiO_2_@Pr-NH_2_@DAP contains elements of C, N, O, Si, and Fe in the structure established by the EDX spectrum (Fig. 5). The Au peak in Fig. 5 is due to the coating of the sample with Au in the procedure of sample preparation for EDX analysis. For further validation, the composition of the as-synthesized catalyst, Elemental mapping of N, Fe, C, Si, and O was performed, confirming the preparation of the nano-catalyst (Fig. 6). According to the results obtained, it can be established that the Fe_3_O_4_@SiO_2_@Pr-NH_2_@DAP, has been effectively synthesized.

### TEM images

**Figure 7:**
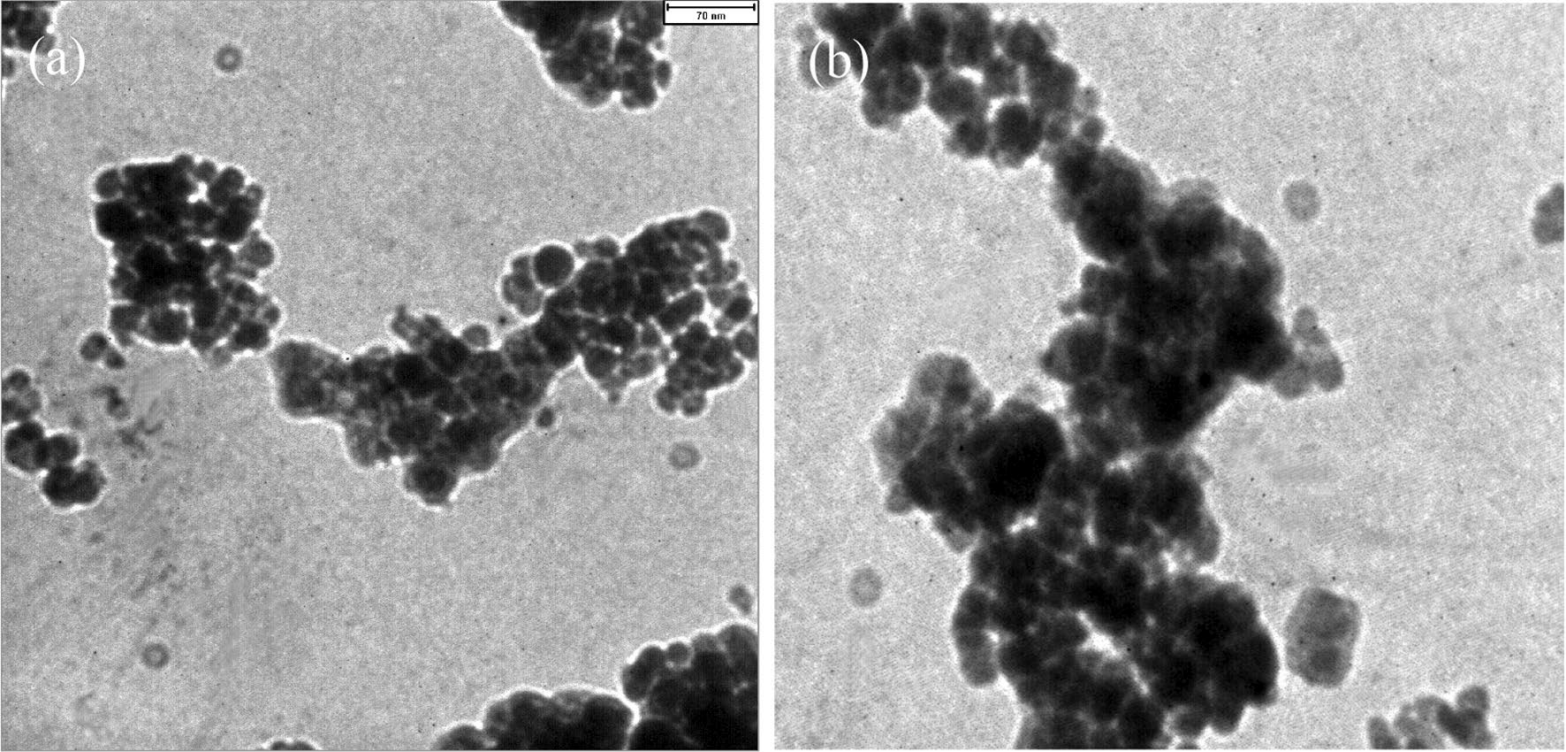
TEM images of Fe_3_O_4_@SiO_2_@Pr-NH_2_@DAP nanoparticles (**a**,**b**).

The TEM image of the magnetic nano-catalyst Fe_3_O_4_@SiO_2_@Pr-NH_2_@DAP in Fig. 7. confirms the core–shell structure well, and it is easy to see that Fe_3_O_4_ nanoparticles are surrounded by a gray shell of SiO_2_, but the magnetic nature of the nanoparticles causes particle aggregation (Fig. 7a,b). The TEM image of MNPs shows that the average size of the synthesized nanoparticles was approximately 22–26 nm and as you can see in the figure, the particles are formed by sticking together.

### X-ray diffraction (XRD) analysis of Fe_3_O_4_@SiO_2_@Pr-NH_2_@DAP

**Figure 8:**
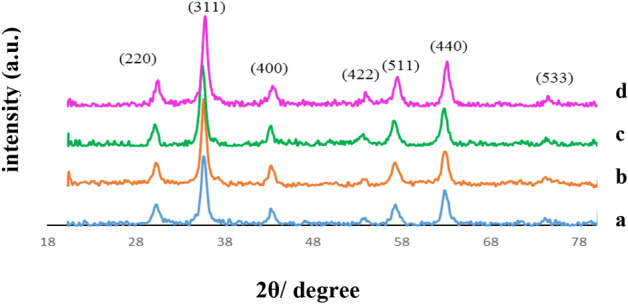
XRD diffraction pattern of (**a**) Fe_3_O_4_MNPs, (**b**) Fe_3_O_4_@SiO_2_, (**c**) Fe_3_O_4_@SiO_2_@Pr-NH_2_ and (**d**) Fe_3_O_4_@SiO_2_@Pr-NH_2_@DAP MNPs.

The crystalline nature of synthesized nano of Fe_3_O_4_ MNPs, Fe_3_O_4_@SiO_2_, Fe_3_O_4_@SiO_2_@Pr-NH_2_, and Fe_3_O_4_@SiO_2_@Pr-NH_2_@DAP was explored by X-ray diffraction (XRD) technique (Fig. 8). The XRD patterns of Fe_3_O_4_ display several characteristic peaks appearing at 2*θ* = 30.52°, 35.82°, 43.44°, 53.91°, 57.84°, 63.14° and 74.54° that were attributed to their crystal planes (1 1 0), (2 2 0), (3 1 1), (4 0 0), (3 3 1), (4 2 2), (5 1 1), (4 4 0) and (5 3 3), of structured magnetite pure Fe_3_O_4_ respectively. It is implicit that they correspond to the crystalline cubic spinel structure, and they agree with (JCPDS card no. 85-1436)^48^. As shown in Fig. 8 of the XRD spectrum of the nanoparticles, the broad reflection around 2θ = 15° to 27° is related to an amorphous silica phase of Fe_3_O_4_@SiO_2_@Pr-NH_2_@DAP MNPs.

### Thermal gravimetric analysis (TGA) and differential thermal analysis (DTA)

**Figure 9:**
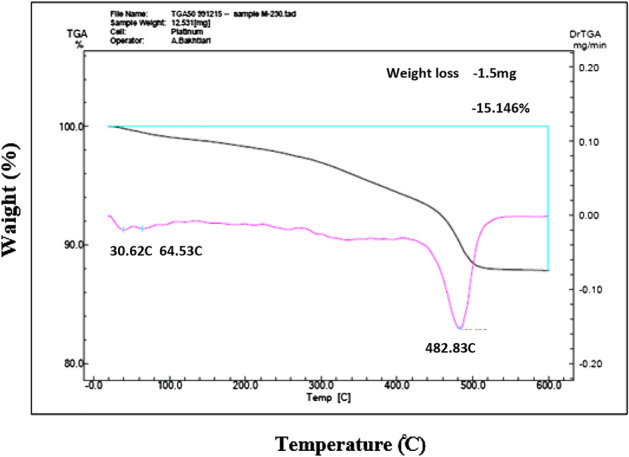
TGA–DTG analysis Fe_3_O_4_@SiO_2_@Pr-NH_2_@DAP nanoparticles.

To study the thermal behaviors of and the presence of stabilized organic ligands on the surface of the synthesized nano-catalyst, analysis, thermogravimetric analysis (TGA), and differential thermal analysis (DTA) used to determine the stability of Fe_3_O_4_@SiO_2_@Pr-NH_2_@DAP NPs was performed in temperature between 25 and 600 °C under inert nitrogen atmosphere (Fig. 9). The thermogram curve Fe_3_O_4_@SiO_2_@Pr-NH_2_@DAP shows two stages of weight loss. The initial weight loss at temperatures up to 200 °C about (1.5%) is generally the removal of surface water adsorbed molecules and surface hydroxyl groups. The small weight loss at second weight loss observed at temperatures between 220 and 600 °C and the main weight loss of the organic grafting is about 15%, which is related to the decomposition of the amino pyrimidine compound deposited on the surface of the magnetic core–shell substrate. Therefore, based on TGA results, it was shown that the compound has high thermal stability, and the functionalization of the nano-catalyst surface with organic groups has been successfully performed.

### VSM

**Figure 10:**
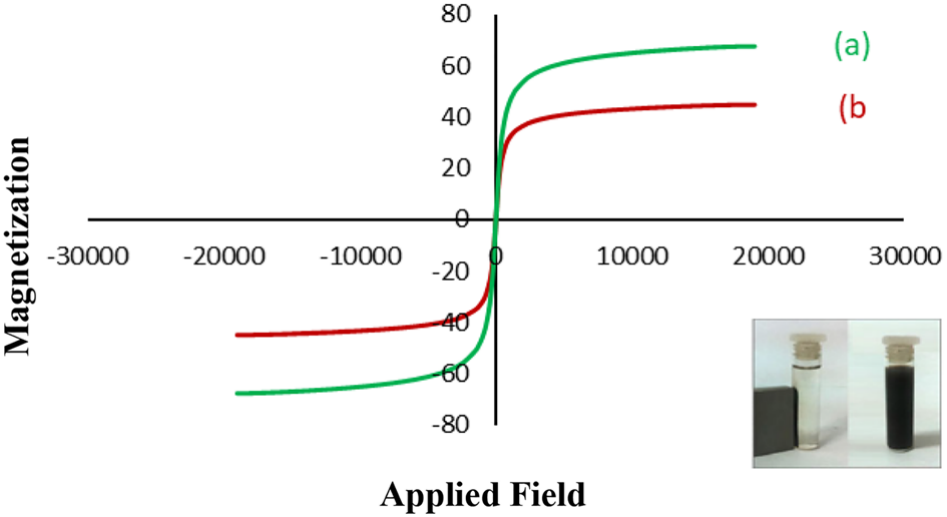
VSM magnetization curves of (**a**) nano-Fe_3_O_4_, (**b**) Fe_3_O_4_@SiO_2_@Pr-NH_2_@DAP nanoparticles.

The magnetic properties of bare Fe_3_O_4_ and coated magnetic nanoparticles were evaluated using a VSM instrument. The magnetization curves of Fe_3_O_4_ (a) and Fe_3_O_4_@SiO_2_@Pr-NH_2_@DAP are shown in Fig. 10. According to the graph, the amount of saturation magnetization (Ms) for bare Fe_3_O_4_ magnetic nanoparticles is 67.53 emu/g and for magnetic Fe_3_O_4_@SiO_2_@Pr-NH_2_@DAP NPs was 44.78 emu/g. is (Fig. 10a,b). The observed decrease in saturation magnetization intensity (Ms), Fe_3_O_4_@SiO_2_@Pr-NH_2_@DAP compared to pure Fe_3_O_4_ nanoparticles due to the presence of silica shell and non-magnetic organic shell around the Fe_3_O_4_ core.

### Determination of the best synthetic pathway for the synthesis of 4*H*-chromans catalyzed by Fe_3_O_4_@SiO_2_@Pr-NH_2_@DAP MNPs

After preparing and characterization of the magnetic nano-catalysts, and due to the pharmaceutical versatilities of the 4*H*-chromane and pyranothiazolopyrimidine derivatives, we encouraged its efficiency as a heterogeneous catalyst was investigated for the synthesis of these heterocyclic scaffolds using an MCR approach.

### Synthesis of pyrano[2,3-*d*]Pyrimidine derivatives catalysed by Fe_3_O_4_@SiO_2_@Pr-NH_2_@DAP MNPs

7-Hydroxy-5*H*-thiazolo[2,3-*a*]pyrimidine-5-one (1 mmol) as heterocyclic 1,3-dione, 4-chloro-benzaldehyde (1 mmol), and malononitrile (1 mmol) were selected as the model reaction, and the effect of different parameters, including solvents, temperature and amount of catalyst was investigated. Outcomes are presented in Table 1. Initially, to demonstrate the type and the amount of catalyst that was investigated in the synthesis of pyranothiazolopyrimidines, the model reaction was performed without catalyst and solvent at 100 °C, which resulted in only a trace amount of the expected product after a prolonged reaction time. Then, the model reaction was explored in the presence of 0.05 g of the synthesized catalyst Fe_3_O_4_@SiO_2_@Pr-NH_2_@DAP, under solvent-free conditions, leading to a 95% yield (Table 1, entry 2). To find the best solvent, the effect of different solvents (including EtOH, H_2_O, EtOH:H_2_O (1:1), toluene, and acetonitrile) and solvent-free condition on the rate and yield of the reaction were investigated (Table 1, entry 3–7). As the results in the table displayed, the solvent-free condition was selected as the most favorable system. In the next step, the model reaction at different amounts of the nano-catalyst and various temperatures was optimized. The highest conversion of 95% in reaction rates (10 min) was reached for 0.05 g catalyst loading at 80 °C (Table 1, entry 9). Finally, to further investigate the catalytic efficiency of the Fe_3_O_4_@SiO_2_@Pr-NH_2_@DAP, the model reaction was accomplished using catalyst components including pure Fe_3_O_4_, Fe_3_O_4_@SiO_2_, Fe_3_O_4_@SiO_2_@Pr-NH_2,_ and the results showed the highest product yield in the presence of Fe_3_O_4_@SiO_2_@Pr-NH_2_@DAP catalyst (Table 1, entry 13–15).

**Table 1:** Screening Optimization of the reaction conditions for the synthesis of pyranothiazolopyrimidine derivative **4a** catalyzed by the Fe_3_O_4_@SiO_2_@Pr-NH_2_@DAP nano-catalyst^a^.

Entry	Solvent^b^	Catalyst amount (g)	Temperature (°C)	Time (min)	Yield (%)
1	Solvent-free	–	100	24 h	Trace
2	Solvent-free	0.05	100	10	95
3	EtOH	0.05	Reflux	100	90
4	H_2_O	0.05	Reflux	100	65
5	EtOH:H_2_O (1:1)	0.05	Reflux	120	80
6	CH_3_CN	0.05	Reflux	120	85
7	Toluene	0.05	100	120	40
8	Solvent-free	0.05	90	10	95
9	Solvent-free	0.05	80	10	95
10	Solvent-free	0.06	80	10	95
11	Solvent-free	0.04	80	30	90
12	Solvent-free	0.04	90	30	92
13^c^	Solvent-free	0.05	80	60	25
14^d^	Solvent-free	0.05	80	60	35
15^e^	Solvent-free	0.05	80	60	55

^a^Reaction conditions: 4‐chlorobenzaldehyde (1 mmol), heterocyclic dione 2b (1 mmol), and malononitrile (1 mmol) were mixed. ^b^Solvent (5 mL). ^c^Catalyst: Fe_3_O_4_, ^d^Fe_3_O_4_@SiO_2_, ^e^Fe_3_O_4_@SiO_2_@Pr-NH_2_.

With the optimized conditions in hand, various aromatic aldehydes bearing electron-withdrawing or electron-releasing groups with two heterocyclic-1,3-diones were tolerated in the same reaction conditions and gave the corresponding products in good to excellent yields (Table 2). Furthermore, when heteroaromatic aldehydes such as pyridine-4-carbaldehyde and thiophene-2-carboxaldehyde were used as substrates, excellent yields of the corresponding products were obtained in a short reaction time (Table 2, entries 4 and 7).

**Table 2:** Synthesis of pyranothiazolopyrimidines **4a**–**4i** by using Fe_3_O_4_@SiO_2_@Pr-NH_2_@DAP nano-catalyst.


Entry	Aldehyde	Dione	Product	Time (min)	Yield (%)	Mp (°C)	
						Found	Reported
1	4-Cl-C_6_H_4_	2b	**4a**	10	95	268–270 (new)	
2	4-Br-C_6_H_4_	2b	**4b**	7	90	264–266 (new)	
3	3,4-di-Cl-C_6_H_3_	2b	**4c**	10	95	278–280 (new)	
4	4-Pyridyl-	2b	**4d**	15	94	268–270 (new)	
5	-C_6_H_5_	2a	**4e**	15	90	257–258	
6	4-Cl-C_6_H_4_	2a	**4f**	10	90	263–265	
7	2-Thienyl-	2a	**4g**	12	93	254–255	
8	4-OMe-C_6_H_4_	2a	**4h**	25	88	226–228	
9	2-Naphthyl	2a	**4i**	20	95	276–278	

### Synthesis of tetrahydro-4*H*-benzopyrans catalyzed by Fe_3_O_4_@SiO_2_@Pr-NH_2_@DAP MNPs (6)

To find the optimal reaction conditions the various solvents, temperatures, types, and amounts of catalyst were investigated, and the results are given in Table 3. Concerning the reaction rates and yields, the best result is achieved using 0.04 g of Fe_3_O_4_@SiO_2_@Pr-NH_2_@DAP MNPs as a catalyst under solvent-free conditions at 70 °C (Table, entry).

**Table 3:** Optimization of the reaction conditions for the synthesis of **6a** catalyzed by Fe_3_O_4_@SiO_2_@Pr-NH_2_@DAP nano-catalyst^a^.

Entry	Solvent^b^	Catalyst amount (g)	Temperature (°C)	Time (min)	Yield (%)
1	Solvent-free	–	100	24 h	Trace
2	Solvent-free	0.05	100	5	95
3	EtOH	0.05	Reflux	50	90
4	H_2_O	0.05	Reflux	100	70
5	EtOH:H_2_O (1:1)	0.05	Reflux	100	85
6	CH_3_CN	0.05	Reflux	100	85
7	Toluene	0.05	100	100	50
8	Solvent-free	0.05	90	5	95
**9**	Solvent-free	0.05	80	5	95
10	Solvent-free	0.07	80	7	90
11	Solvent-free	0.04	80	5	95
12	Solvent-free	0.04	70	6	95
13^c^	Solvent-free	0.04	70	50	30
14^d^	Solvent-free	0.04	70	50	45
15^e^	Solvent-free	0.04	70	50	60

^a^Reaction conditions: 4‐chlorobenzaldehyde (1 mmol), dimedone (1 mmol), and malononitrile (1 mmol) were mixed. ^b^Solvent (5 mL). ^c^Catalyst: Fe_3_O_4_, ^d^Fe_3_O_4_@SiO_2_, ^e^Fe_3_O_4_@SiO_2_@Pr-NH_2_.

Likewise, different types of aromatics and heteroaromatic aldehydes have been examined using the optimal reaction conditions for the desired products in good to excellent yields (Table 4).

**Table 4:** Synthesis of 4*H*-pyrans **6a**–**k** using Fe_3_O_4_@SiO_2_@Pr-NH_2_@DAP nano-catalyst.


Entry	Aldehyde	Dione	Product	Time (min)	Yield (%)	Mp (°C)	
						Found	Reported
1	C_6_H_5_	Dimedone	**6a**	15	93	226–228	226–228^49^
2	4-Cl-C_6_H_4_	Dimedone	**6b**	5	95	207–209	208–210^50^
3	4-Br-C_6_H_4_	Dimedone	**6c**	7	96	211–212	207–208^50^
4	4-NO_2_-C_6_H_4_	Dimedone	**6d**	7	94	179–181	178–179^49^
5	3,4-di-Cl-C_6_H_3_	Dimedone	**6e**	5	95	183–184	184–186^51^
6	2,6-di-Cl-C_6_H_3_	Dimedone	**6f**	10	92	247–249	249–251^52^
7	4-isopropyl-C_6_H_4_	Dimedone	**6g**	20	93	206–207	203–207^53^
8	4-CN-C_6_H_4_	Dimedone	**6h**	15	92	227–229	226–228^54^
9	3-OH-C_6_H_4_	Dimedone	**6i**	40	94	232–234	231–234^53^
10	2-Thienyl	Dimedone	**6j**	15	85	223–225	223–237^54^
11	5-Br-2-HO-C_6_H_3_	Dimedone	**6k**	20	95	192–194	190–193^55^

The melting point of the synthesized compounds was compared with the previously reported cases and confirmed. The chemical structure of several compounds was selectively characterized using detection techniques such as FT-IR, ^1^HNMR, ^13^CNMR, and mass. For example, FT-IR spectrum of the compound of 8-amino-6-(4-chlorophenyl)-5-oxo-5*H*,6*H*-pyrano[3,2-*d*]thiazolo [2,3-*a*]pyrimidine-7-carbonitrile showed the absorptions peaks at 3322, and 3179 cm^−1^ (NH_2_), 2204 cm^−1^ (CN), 1684 cm^−1^ (C=O) and 1665 cm^−1^ (C≡N) confirmed the successful synthesis of **4a**.

Also, the ^1^HNMR spectrum exhibited a singlet for CH methine with one proton (δ = 4.57), a triplet for NH_2,_ aromatic protons with three protons (δ = 7.28), and a doublet for aromatic protons with two protons (δ = 7.35). The CH–S and CH–N of the thiazole ring resonated at δ = 7.53 and 7.59 ppm (a doublet peak, 1H), respectively. The ^1^H-decoupled ^13^C NMR spectrum of **4a** showed 14 distinct resonances, consistent with the proposed structure. The mass spectrum of **4a** exhibited the molecular ion peak at m/z 399 according to the mass of the suggested product.

### Reaction mechanism

**Figure 11:**
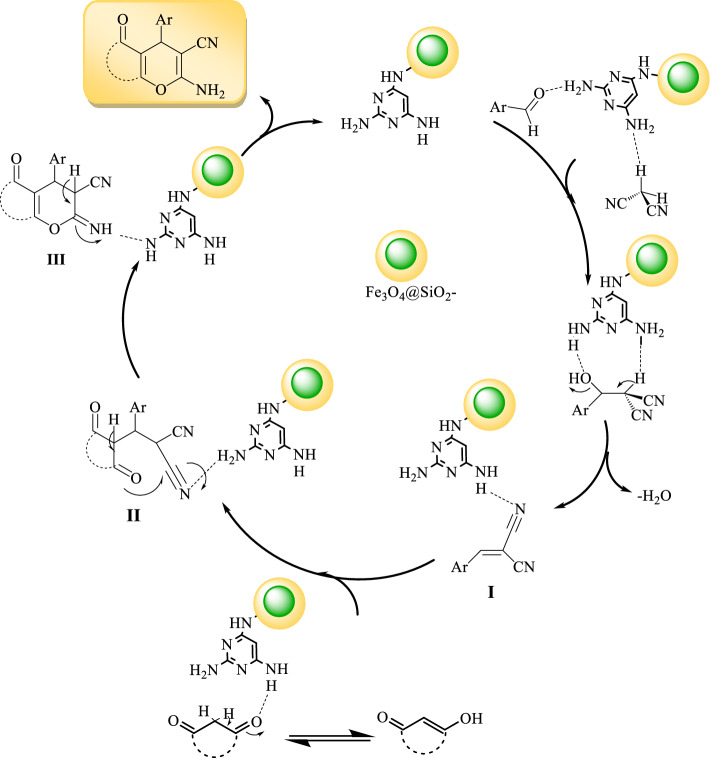
Mechanistic rationalization for the synthesis of expected products **4** and **6**.

The exact mechanism of this synthetic method catalyzed by Fe_3_O_4_@SiO_2_@Pr-NH_2_@DAP NPs composite system is not clear. According to the reaction mechanisms proposed in the literature^52^, the probable mechanism for the preparation of products in the presence of Fe_3_O_4_@SiO_2_@Pr-NH_2_@DAP NPs was outlined in Fig. 11. First of all, maybe Fe_3_O_4_@SiO_2_@Pr-NH_2_@DAP NPs coordinates with an aromatic aldehyde to increase its electrophilic character of it and then malononitrile reacts very fast with an aldehyde to give the Knoevenagel condensation product (I) with loss of water. Then, 1,3-dione (3) is tautomerized to enol form which is activated by the interaction of the nano-catalyst with its carbonyl group, and subsequently nucleophilic attack to [I] and afforded the Michael adduct (II). Followed by intramolecular nucleophilic cyclization and intermediate (III) is obtained, which subsequently undergoes tautomerization to produce the desired product.

### Recoverability and regeneration of the nano-catalyst

**Figure 12:**
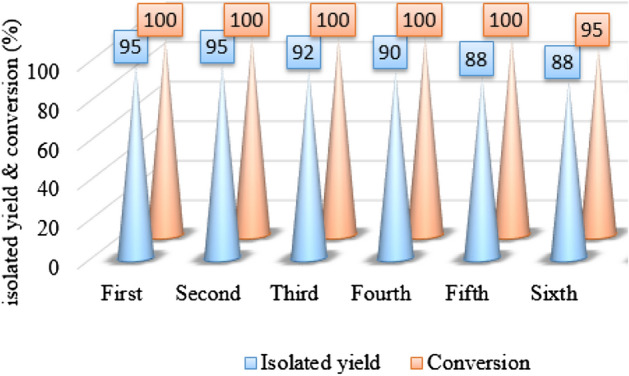
Recoverability of Fe_3_O_4_@SiO_2_@Pr-NH_2_@DAP nano-catalyst in the synthesis of compound **6b**.

**Figure 13:**
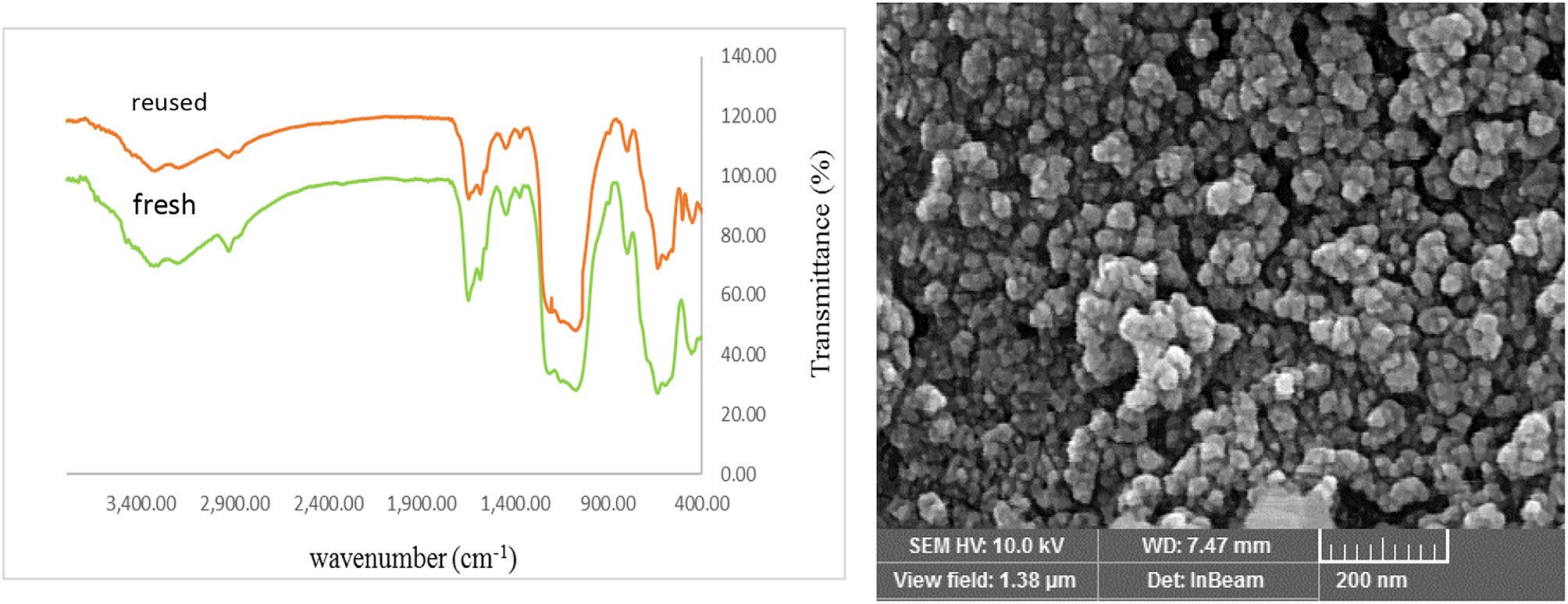
FT-IR spectrum (red spectrum) and FE-SEM image of the recovered catalyst.

From the perspective of green chemistry, the reuse and recovery of catalysts are important aspects of magnetic catalysts examined in this study. For this purpose, the recovery of the nano-catalyst in the model reaction was studied under optimal conditions. At the end of the reaction, the reaction was stopped. Hot ethanol was added to the reaction mixture. The catalyst was collected using a magnetic field, washed several times with ethanol and water, and dried at 60 °C in an oven. Then the recovered nano-catalyst was used for six consecutive cycles under the same reaction conditions. As shown in Figs. 12 and 13, the synthesis catalyst without a negligible decrease in its catalytic efficiency can be reused.

After six runs, the recovered catalyst had no distinct change in structure, as evident from a comparison of its FT-IR spectrum with that of the fresh catalyst. Also, the FE-SEM image of the recovered catalyst shows that the morphology of the reused catalyst was restored without any obvious changes after six catalytic runs (Fig. 13).

### Comparison of the catalyst

To further evaluate the catalysts synthesized in this study, we have compared the performance of Fe_3_O_4_@SiO_2_@Pr-NH_2_@DAP MNPs with reported catalysts for the reaction of 4-chloro-benzaldehyde, dimedone, and malononitrile in the preparation of 2-amino-4-(4-chlorophenyl)-7,7-dimethyl-5-oxo-8,7,6,5-Tetrahydro-4*H*-chroman-3-carbonitrile.

As the results of Table 5 show, that some catalysts have synthesized a high-efficiency product. Still, they have disadvantages such as long reaction time, the toxicity of the catalyst, and some others not possible to recover the catalyst. In the current method, Fe_3_O_4_@SiO_2_@Pr-NH_2_@DAP MNPs a green, non-toxic, and highly recyclable catalysts according to the green chemistry perspective.

**Table 5:** Comparison of the results of Fe_3_O_4_@SiO_2_@Pr-NH_2_@DAP MNPs with reported catalysts in literature.

Entry	Catalyst	Reaction condition	Time (min)	Yield (%)	References
1	Fe_3_O_4_@MCM41@Zr-piperazine-MNPs	EtOH:H_2_O/75 °C	40	85	^56^
2	RE(POF)_3_ (5 mol%)	EtOH/60 °C	300	93	^57^
3	Mg(ClO_4_)_2_ (25% w)	EtOH/reflux	180	90	^58^
5	I_2_ (mol%10)	DMSO/120	240	90	^59^
6	SB-DABCO (0.06 g)	H_2_O:EtOH/24	20	90	^60^
7	Piperazine (15 mol%)	Solvent free/24	27	92	^61^

## Conclusion

In summary, a suitable and effective method was introduced for the synthesis of 2-amino-3-cyano-4*H*-chromenes through a one-pot three-component reaction of aromatic and heteroaromatic aldehydes, malononitrile, and various 1,3-diones such as dimedone and heterocyclic 1,3-diones using Fe_3_O_4_@SiO_2_@Pr-NH_2_@DAP core–shell nanocomposite as a green and environmentally friendly catalyst. The structural properties of Fe_3_O_4_@SiO_2_@Pr-NH_2_@DAP MNPs were evaluated by different methods such as FT-IR, EDX, FE-SEM, TEM, XRD, and VSM analysis. Furthermore, the advantages of the proposed approach include short reaction time, good to high yields of the products, use of commercially available and cheap, and high efficiency of the catalyst under mild and operational simplicity. The facile reusability of the catalyst and solvent-free conditions of this reaction are also other unique features of this research, which are very important in green chemistry.

### Supplementary Information

Supplementary Information.

## Data Availability

The Supporting Information is available, and in it, experimental procedures and characterization of all new compounds obtained, including ^1^H and ^13^C NMR, Mass, and FT-IR spectra, are available.
