# Supplementary material for: Synthesis of Fe3O4@SiO2@Pr-NH2@DAP as a magnetic recyclable nano-catalyst for efficient synthesis of pyranothiazolopyrimidines and 4H-pyrans under solvent-free condition
Source: Sci Rep. 2023 Sep 11;13:14937. doi: 10.1038/s41598-023-41793-z (PMC10495395; doi:10.1038/s41598-023-41793-z)
Supplement: Supplementary file 1 — Supplementary Information. [file 41598_2023_41793_MOESM1_ESM.pdf]

# Supporting Information


Experimental **1**

Spectra and physical data of **4a-6k** **1-3**

Copies of $^{1}$H NMR, $^{13}$C NMR, IR and Mass **4-19**


**Spectra and physical data of 4a-6k**

**8-amino-6-(4-chlorophenyl)-5-oxo-5*H*,6*H*-pyrano[2,3-*d*]thiazolo[3,2-*a*]pyrimidine-7-carbonitrile(4a):**

(0.34 g, 95%); white solid; mp=268-270 ºC (from EtOH); FT-IR (KBr): $\nu_{max}$/cm$^{-1}$ 3324, 3179 ($NH_2$), 2204 (CN), 1683 (C=O), 1664(C=N) $^{1}$HNMR (300.13 MHz, DMSO-$d_6$): δ (ppm) 7.92 (1H, J= 1.2 Hz, Ar-H ), 7.54 (1H, d, J= 4.5 Hz, Ar-H ), 7.37(2H, d, J= 7.8 Hz, Ar-H ), 7.29 (3H, t, J= 4.5, $NH_2$, Ar-H ), 4.57 (s, 1 H, CH); $^{13}$C NMR: δ C (75.46 MHz, DMSO- d6) 162.1, 159.6, 158.0, 157.3, 143.5, 131.7, 129.8, 128.7, 122.2, 119.9, 114.1, 95.0, 57.2, 36.8; MS: (m/z, %): m/z 356(M, 15), 353( M, 28), 289 (15), 264 (18), 241 (92), 167(88), 125 (90), 99 (90), 57(90), 44 (88), 28(100).

**8-amino-6-(4-bromophenyl)-5-oxo-5*H*,6*H*-pyrano[2,3-*d*]thiazolo[3,2-*a*]pyrimidine-7-carbonitrile(4b):**

(0.36 g, 90%); white solid; mp= 264-266 ºC (from EtOH); FT-IR (KBr): $\nu_{max}$/cm$^{-1}$ 3321, 3178 ($NH_2$), 2204 (CN), 1685 (C=O), 1664(C=N) $^{1}$HNMR (300.13 MHz, DMSO-$d_6$): δ (ppm) 7.92 (1H, J= 4.8 Hz, Ar-H ), 7.48-7.54 (3H, m, Ar-H ), 7.37 (2H, s, $NH_2$ ), 7.21 (2H, d, J= 8.1 Hz, Ar-H ), 4.56 (s, 1 H, CH); $^{13}$C NMR: δ C (75.46 MHz, DMSO- d6) 162.1, 159.6, 158.8, 157.4, 143.9, 131.6, 130.2, 122.2, 120.3, 120.0, 114.1, 94.9, 57.2, 36.2; MS: (m/z, %): m/z 401 (M, 28), 402( M $^{+1}$, 28), 334 (10), 243(100), 150 (25), 127(88), 102 (22), 75 (36), 58(80), 44 (45), 28(100).

**8-amino-6-(3,4-dichlorophenyl)-5-oxo-5*H*,6*H*-pyrano[2,3-*d*]thiazolo[3,2-*a*]pyrimidine-7-carbonitrile (4c):**

(0.36 g, 93%); white solid; mp= 278-280 ºC (from EtOH); FT-IR (KBr): $\nu_{max}$/cm$^{-1}$ 3315, 3173 ($NH_2$), 2202 (CN), 1686 (C=O), 1665(C=N) $^{1}$HNMR (300.13 MHz, DMSO-$d_6$): δ (ppm) 7.93 (d, J= 4.5 Hz, Ar-H), 7.51-7.59 (m, 3H, Ar-H), 7.33 (s, 2H, $NH_2$), 7.25 (dd, J= 8.4, 2.4 Hz, Ar-H), 4.63 (s, 1 H, CH); $^{13}$C NMR: δC (75.46 MHz, DMSO- d6) 162.3, 159.6, 158.8, 157.4, 145.6, 131.2, 130.9, 130.1, 129.8, 128.5, 122. 3, 119.8, 114.2, 94.3, 56.7, 36.7. MS: (m/z, %): m/z 389 (M, 10), 322 (10), 265 (5), 243 (M, 100), 168 (22), 126 (63), 99 (55), 103 (20), 57 (55), 28 (77).

**8-amino-5-oxo-6-(pyridin-4-yl)-5*H*,6*H*-pyrano[2,3-*d*]thiazolo[3,2-*a*]pyrimidine-7-carbonitrile(4d):**
(0.30 g, 94%); white solid; mp= 268-269 ºC (from EtOH); FT-IR (KBr): $\nu_{max}/cm^{-1}$ 3314, 3173 ($NH_2$), 2204 (CN), 1679 (C=O), 1664(C=N) $^1$HNMR (300.13 MHz, DMSO-$d_6$): δ (ppm) 8.49 (d, *J*= 6.3 Hz, 2H, Ar-H), 7.95 (d, J= 6.0 Hz,1H, Ar-H), 7.55 (d, *J*= 5.1 Hz,1H, Ar-H), 7.36 (s, 2 H, $NH_2$), 4.60 (s, 1 H, CH); $^{13}$C NMR: δ C (75.46 MHz, DMSO- d6) 162.4, 159.8, 159.1, 157.4, 152.8, 150.1, 123.2, 122.3, 119.8, 114.2, 94.0, 32.24, 56.30, 36.85; MS: (m/z, %): m/z 323(M, 5), 321 (30), 256 (25), 243 (M, 5), 127 (90), 103 (20), 66 (35), 51 ( 82), 29 (100).

**8-amino-5-oxo-6-phenyl-2,3-dihydro-5*H*,6*H*-pyrano[2,3-*d*]thiazolo[3,2-*a*]pyrimidine-7-carbonitrile(4e):**

(0.3g, 90%), white powder; mp= 257–258 °C, IR (KBr, $cm^{-1}$): $\nu_{max}/cm^{-1}$= 3314, 3167 ($NH_2$), 2167(C=N), 1680 (C=O), 1539 (C=N); $^1$H NMR (300 MHz, DMSO-$d_6$): δ 7.29–7.34 (m, 2H, Ar-H), 7.16–7.25 (m,5H, $NH_2$, Ar-H), 4.39 (s, 1H, CH), 4.22–4.35 (m, 2H, $CH_2$–N), 3.52 (t, 2H, *J*= 7.6 Hz, $CH_2$–S),; $^{13}$C NMR (75 MHz, DMSO-$d_6$): δ 27.2, 37.1, 49.3, 57.7, 98.0, 120.1, 127.3, 127.9, 128.8, 144.5, 159.6, 159.6, 159.7, 165.3; MS: (m/z, %) 324 ( M+, 9), 244 (100), 128 (74), 101 (62), 85 (78), 43 (44).

**2-amino-4-(4-chlorophenyl)-7,7-dimethyl-5-oxo-5,6,7,8-tetrahydro-4*H*-chromene-3-carbonitrile(6b):**

(0.31 g, 95%); white solid; mp = 207-209 ºC (from EtOH) (Lit.[136] 223–227 ºC); FT-IR (KBr): $\nu_{max}/cm^{-1}$ 3347, 3172 ($NH_2$), 2958 ($sp^3$ C-H), 2189 (CN), 1684 (C=O).

**2-amino-7,7-dimethyl-4-(4-nitrophenyl)-5-oxo-5,6,7,8-tetrahydro-4*H*-chromene-3-carbonitrile(6d):**

(0.32 g, 95%); white solid; mp =179-181 ºC (from EtOH) (Lit.[138] 223–227 ºC); FT-IR (KBr): $\nu_{max}/cm^{-1}$ 3407, 3319 ($NH_2$), 3177 (C-H, aromatic), 2978($sp^3$ C-H), 2184 (CN), 1672 (C=O), MS: (m/z, %): m/z 338 (M, 5), 336 (100), 215 (100), 160 (65), 82 ( 36), 66 (65), 28 (100).

**2-amino-4-(3,4-dichlorophenyl)-7,7-dimethyl-5-oxo-5,6,7,8-tetrahydro-4*H*-chromene-3-carbonitrile(6e):**

(0.32 g, 95%); white solid; mp =183-184 ºC (from EtOH) (Lit.[139] 183-186ºC); FT-IR (KBr): $\nu_{max}/cm^{-1}$ 3379, 3183 ($NH_2$), 3177 (C-H, aromatic), 2958($sp^3$ C-H), 2188 (CN), 1676 (C=O).

**2-amino-4-(4-isopropylphenyl)-7,7-dimethyl-5-oxo-5,6,7,8-tetrahydro-4*H*-chromene-3-carbonitrile(6g):**

(0.31 g, 92%);white solid ; mp = 206-207 ºC (from EtOH) (Lit.[141] 206–208 ºC); FT-IR (KBr): $\nu_{max}/cm^{-1}$ 3322, 3179 ($NH_2$), 3207 (C-H, aromatic), 2962 ($sp^3$ C-H), 2204 (CN), 1684 (C=O), $^1$H NMR

(300.13 MHz, DMSO-$d_6$): δ (ppm) 7.18 (d, J= 7.5Hz, 2H, Ar-H), 7.5 (d, *J*= 8.2Hz, 2H, Ar-H), 7.00 (s, 2 H, $NH_2$), 4.15 (s, 1 H, CH), 2.8-2.9 (m, 1 H, CH), 2.09 (2H, brs, $CH_2$), 2,19 (2H, AB system, *J* =15.3 Hz, $CH_2$-CO), 1.18 (d, *J* =6.6 Hz, 6H, $2CH_3$-CH), 1.06 (s, 3 H, $CH_3$), 0.99 (s, 3 H, $CH_3$) ; $^{13}C$ NMR: δ C (75.46 MHz, DMSO-$d_6$) 196.1 (C=O), 162.9, 158.9, 146.9, 142.65, 127.45, 126.74, 120.32, 113.27, 58.96, 50.47, 40.14, 35.60, 33.49, 32.32, 28.86, 27.38, 24.40, 24.31.

**2-amino-4-(4-cyanophenyl)-7,7-dimethyl-5-oxo-5,6,7,8-tetrahydro-4*H*-chromene-3-carbonitrile(6h):**

0.3 g, 94%); white solid; mp =227-229 ºC (from EtOH) (Lit.[142] 226–228 ºC); FT-IR (KBr): $\nu_{max}/cm^{-1}$ 3354, 3326 ($NH_2$), 3177 (C-H, aromatic), 2963($sp^3$ C-H), 2227, 2194 (2CN), 1686 (C=O), 1674(C=N).

**2-amino-4-(3-hydroxyphenyl)-7,7-dimethyl-5-oxo-5,6,7,8-tetrahydro-4*H*-chromene-3-crbonitrile (6i):**

(0.26g, 85%); white solid; mp = 232-234ºC (from EtOH) (Lit.[251] 231-234ºC); FT-IR (KBr): νmax/$cm^{-1}$ 3312, 3174 ($NH_2$), 3177 (C-H, aromatic), 2960(sp3 C-H), 2250 (CN), 1702 (C=O), 1641(C=N).

**2-Amino-7,7-dimethyl-5-oxo-4-(thiophen-2-yl)-5,6,7,8-tetrahydro-4*H*-chromene-3-carbonitrile (6j):**

(0.28 g, 95%); Yellow solid; mp= 223-225 ºC (from EtOH) (Lit.[136] 223–227 ºC); FT-IR (KBr): $\nu_{max}/cm^{-1}$ 3382, 3321 ($NH_2$), 3207 (C-H, aromatic), 2962 ($sp^3$ C-H), 2198 (CN), 1658 (C=O), $^1H$ NMR (300.13 MHz, DMSO-$d_6$): δ (ppm) 7.35 (d, *J*= 4.5 Hz, 1H, Ar-H), 7.16 (s, 2 H, $NH_2$), 6.87-6.94 (m, 2 H, Ar-H), 4.55 (s, 1 H, CH), 2.5 (m, 2 H, $CH_2$), 2.14-2.36 (2H, AB system, *J*= 16.2 Hz, $CH_2$), 1.07 (s, 3 H, $CH_3$), 1.00 (s, 3 H, $CH_3$) ; $^{13}C$ NMR: δ C (75.46 MHz, DMSO- $d_6$) 196.0 (C=O), 162.9, 159.3, 149.7, 127.2, 124.9, 124.4, 120.0, 113.3, 58.5, 50.3, 40.0, 32.2, 30.8, 29.1, 26.9; MS: (m/z, %): m/z 300 ($M^+$, 5%), 298 (M, 100), 265 (M, 5), 232 (5), 215 (44), 160 (20), 133 (15), 82 ( 20), 66 (25).

**2-amino-4-(5-bromo-2-hydroxyphenyl)-7,7-dimethyl-5-oxo-5,6,7,8-tetrahydro-4*H*-chromene-3-carbonitrile(6k):**

(0.34 g, 88%); white solid; mp= 192-194ºC (from EtOH) (Lit.[144] 191-193ºC); FT-IR (KBr): $\nu_{max}/cm^{-1}$ 3312, 3174 ($NH_2$), 3177 (C-H, aromatic), 2960($sp^3$ C-H), 2250 (CN), 1702 (C=O), 1641(C=N).

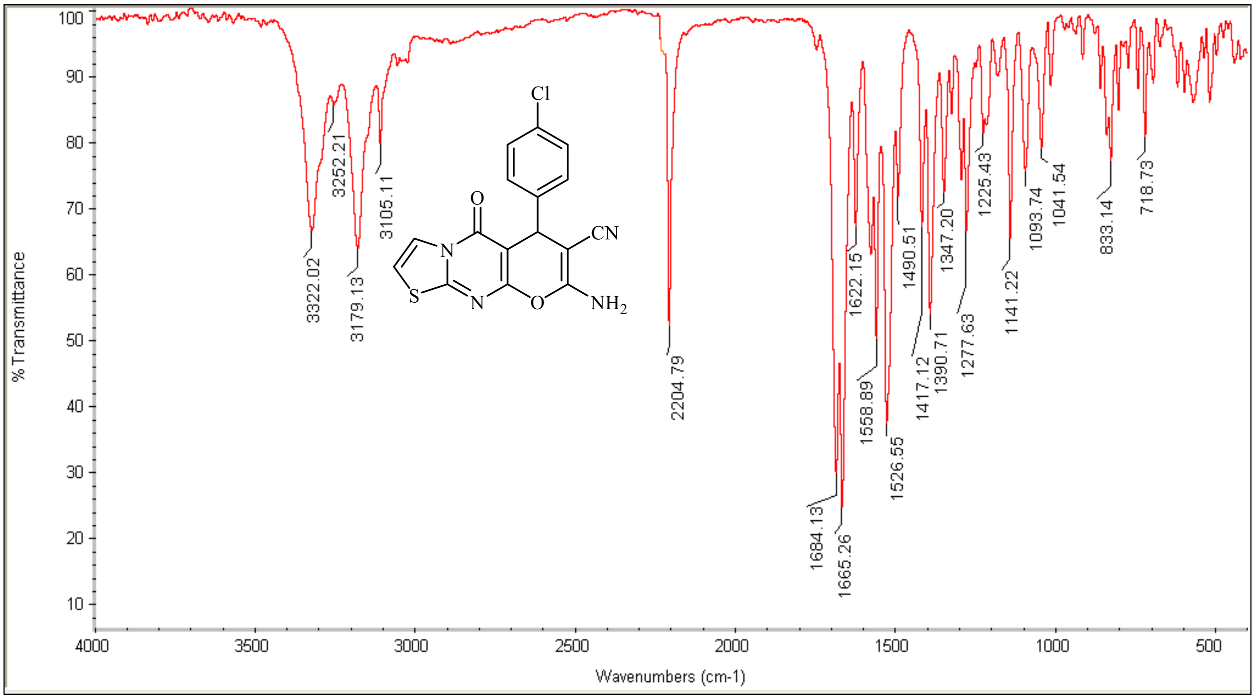


IR spectra of compound **4a**

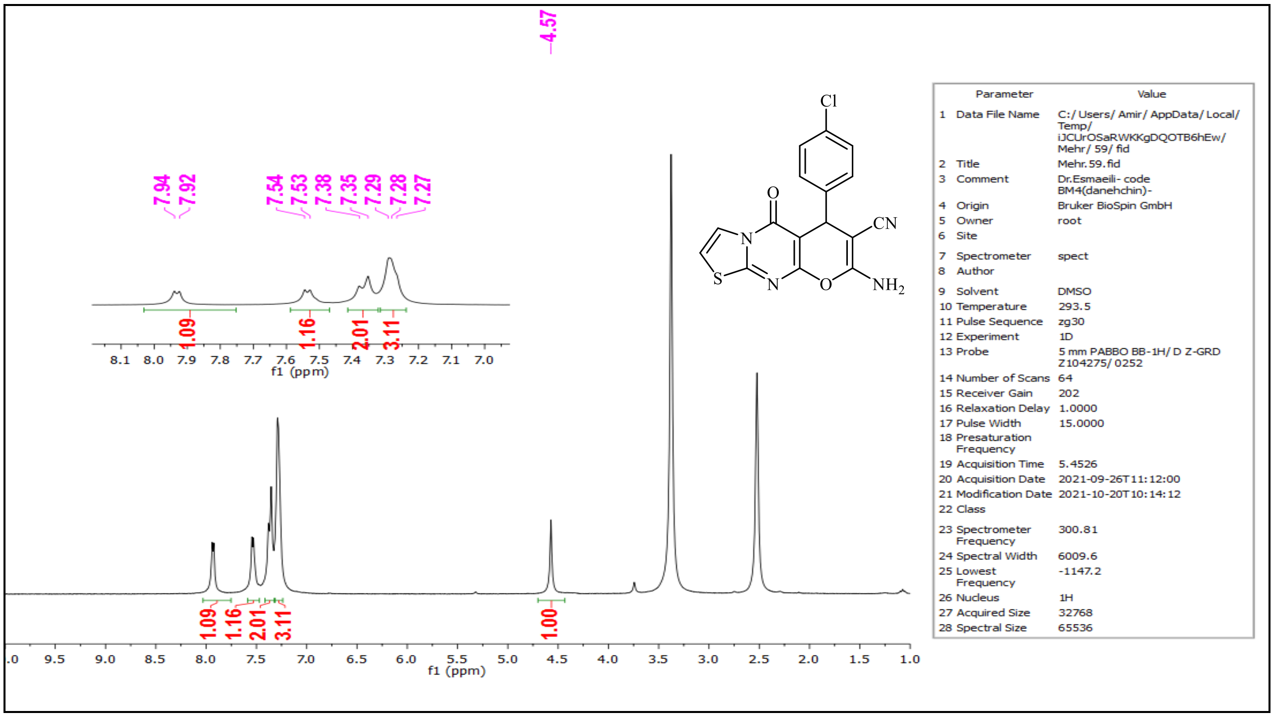

$^{1}$H NMR spectra of compound **4a**

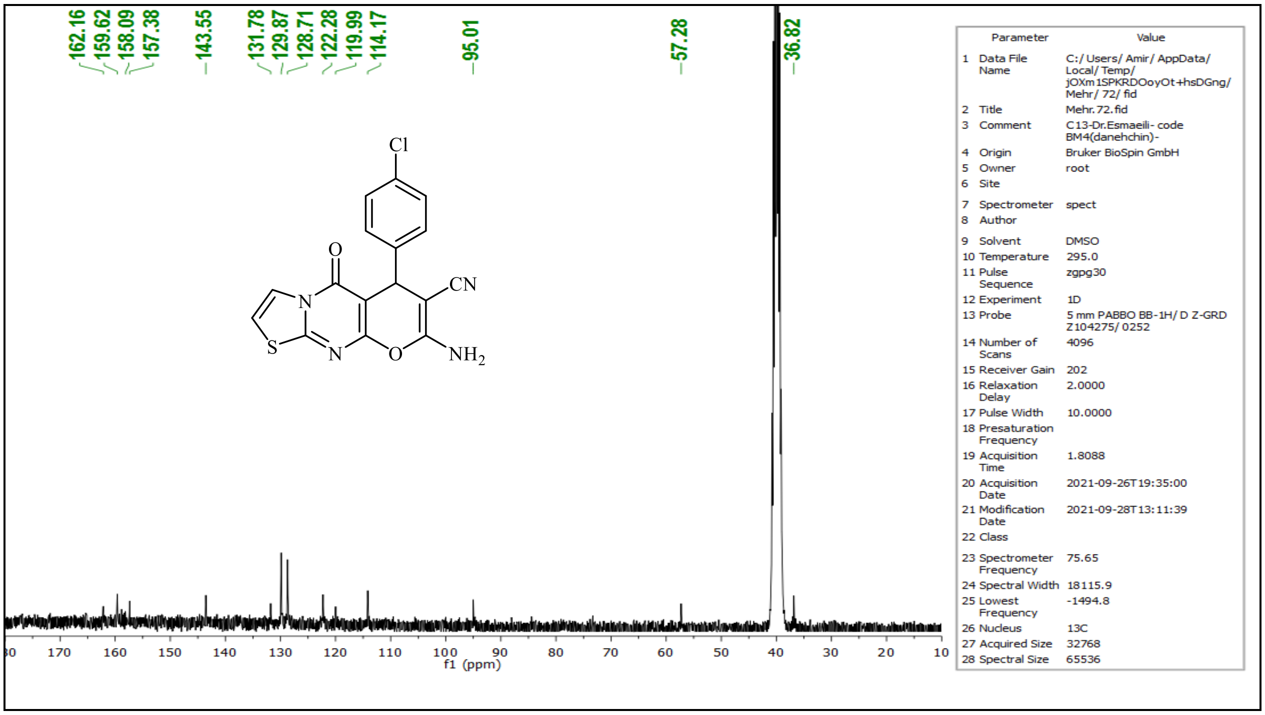


$^{13}$C NMR spectra of compound **4a**

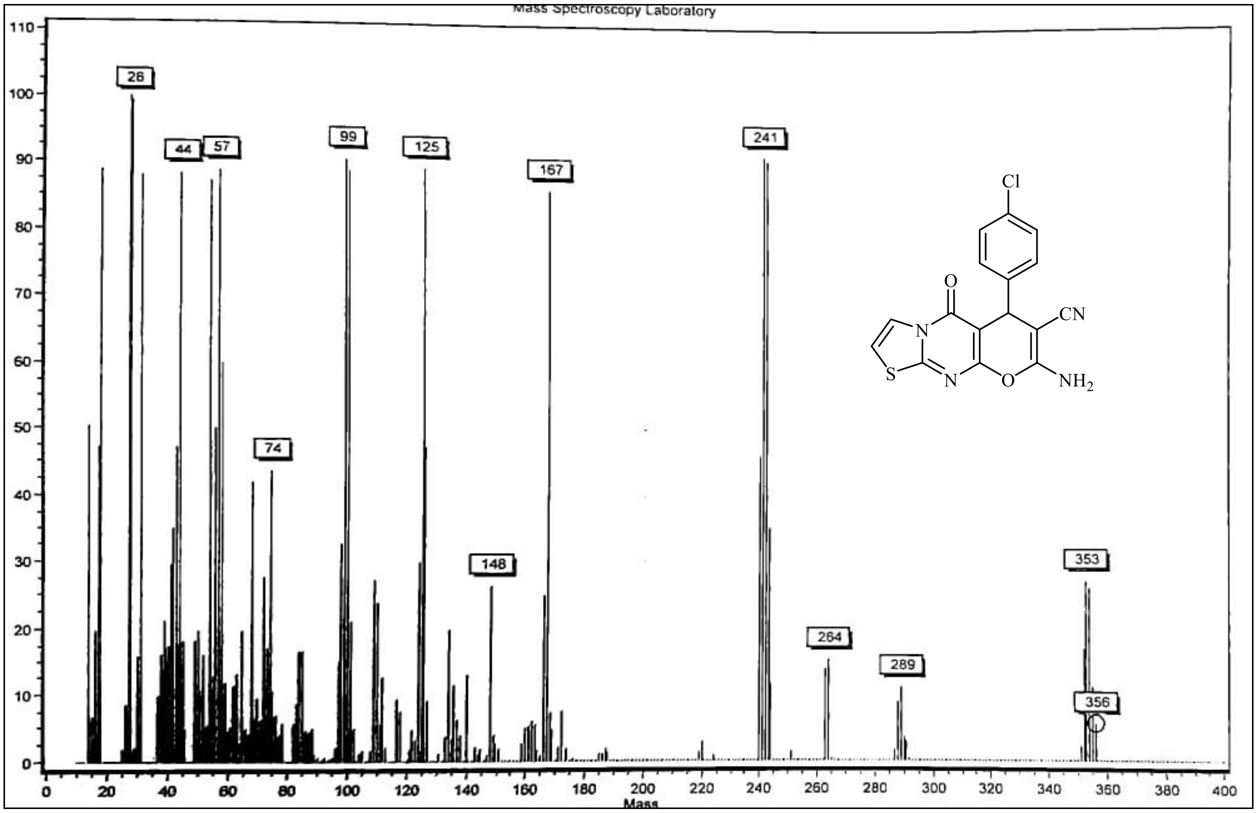


Mass spectra of compound **4a**

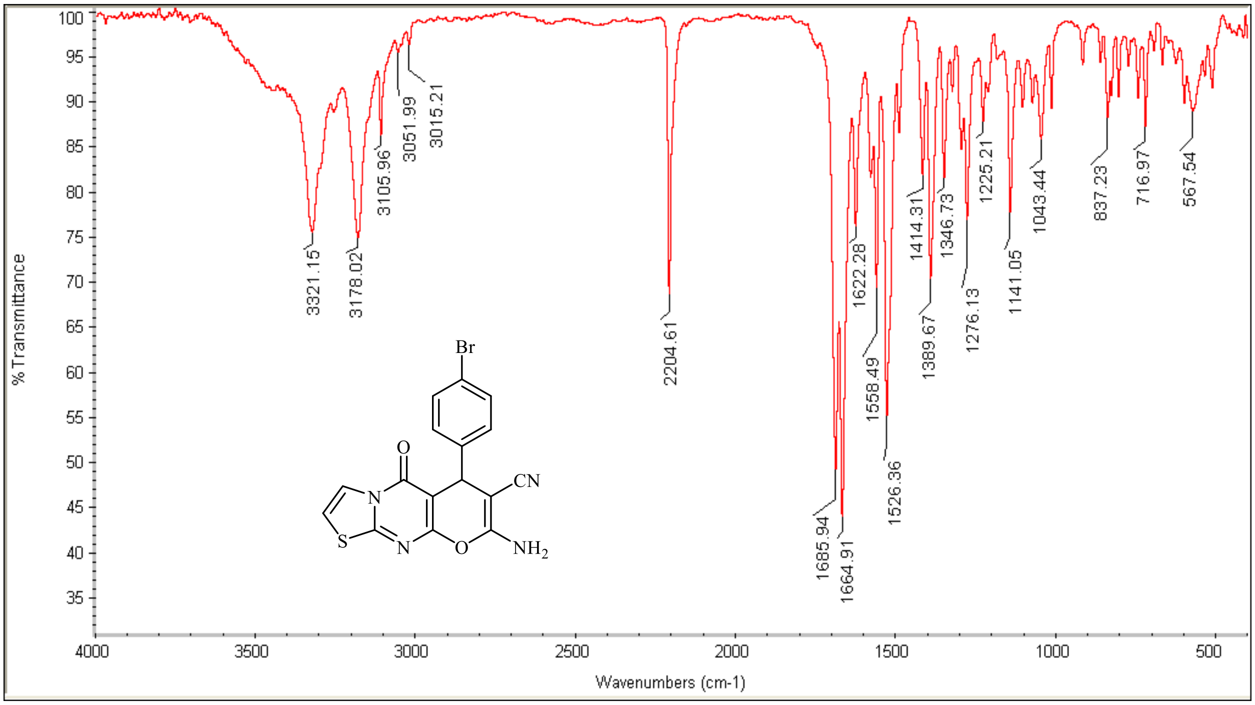


IR spectra of compound **4b**

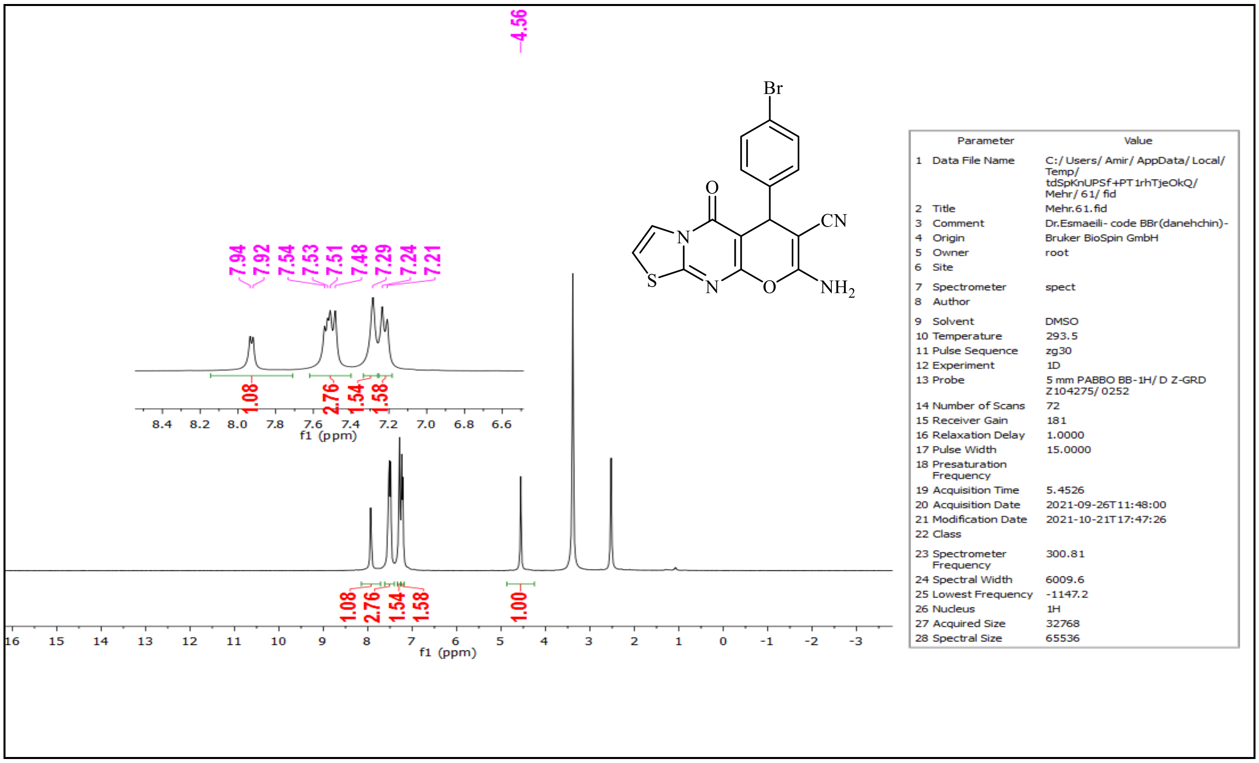


$^{1}$H NMR spectra of compound **4b**

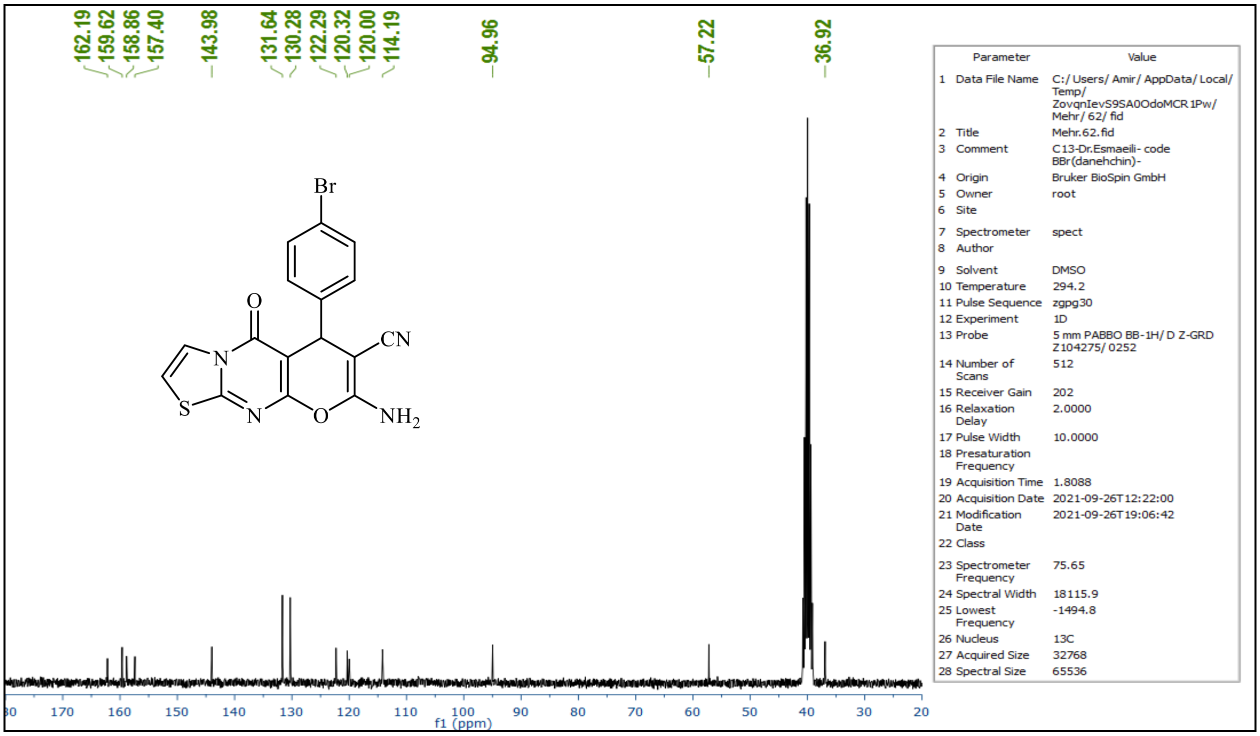


$^{13}$C NMR spectra of compound **4b**

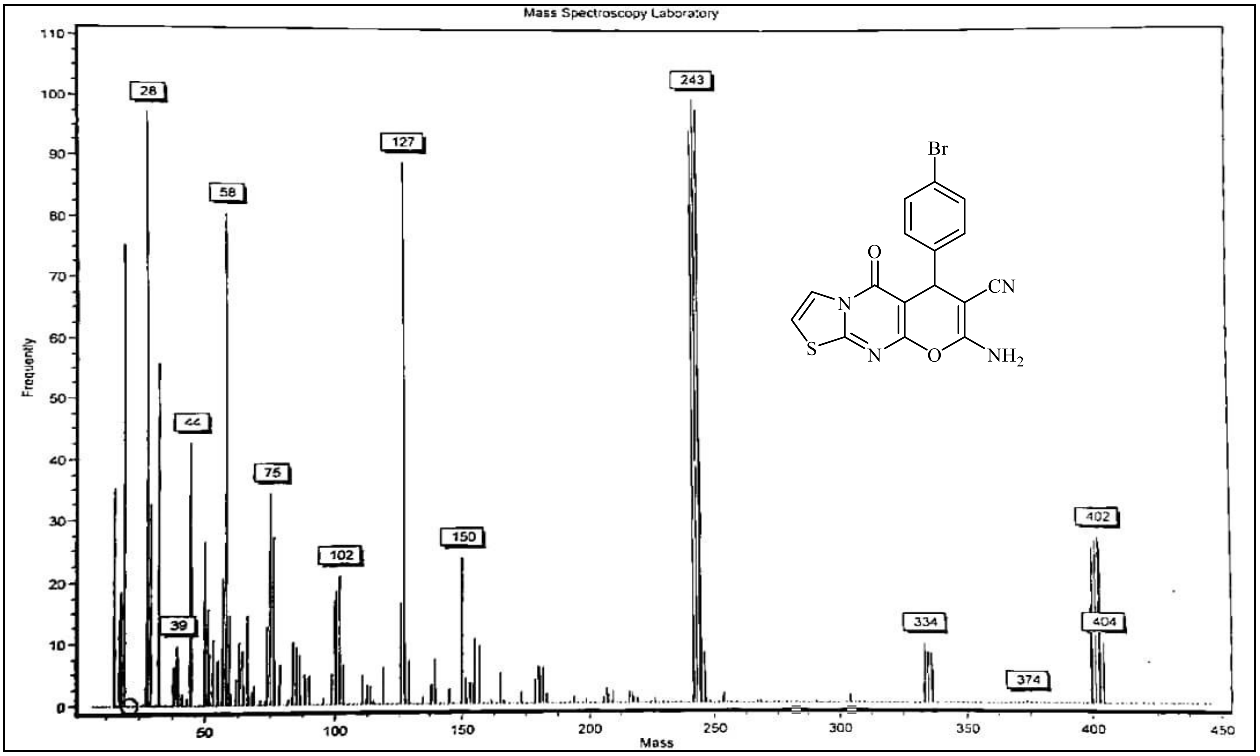


Mass spectra of compound **4b**

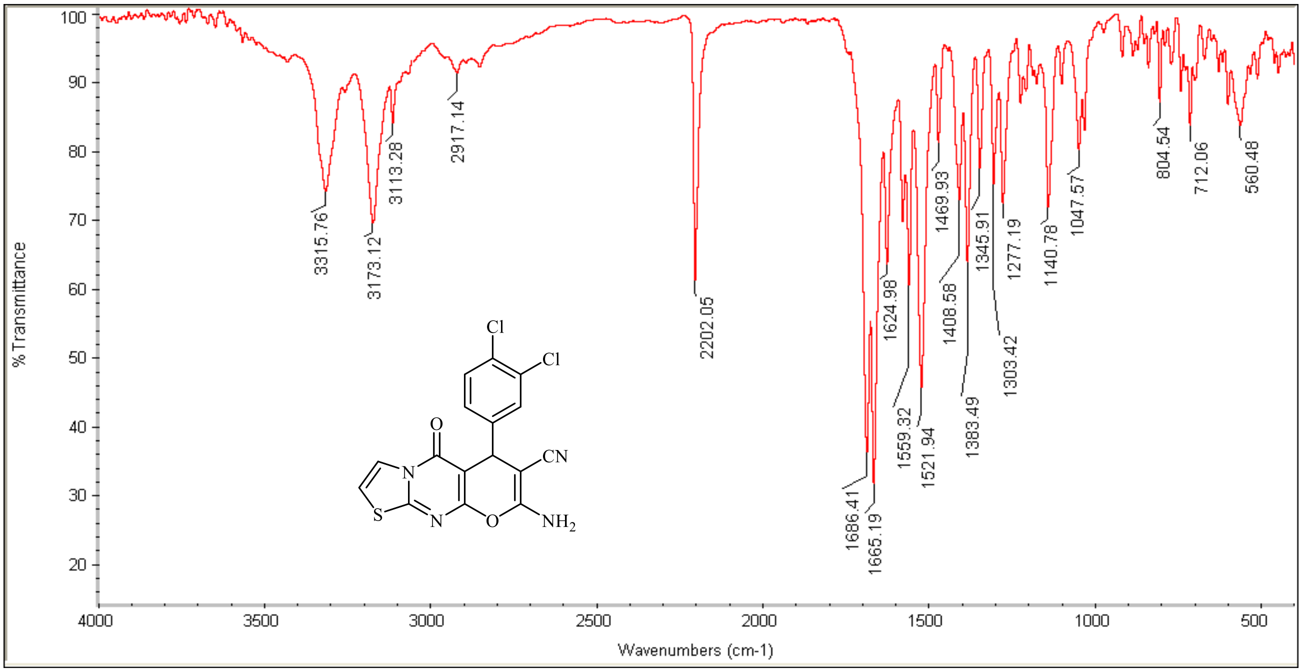


IR spectra of compound **4c**

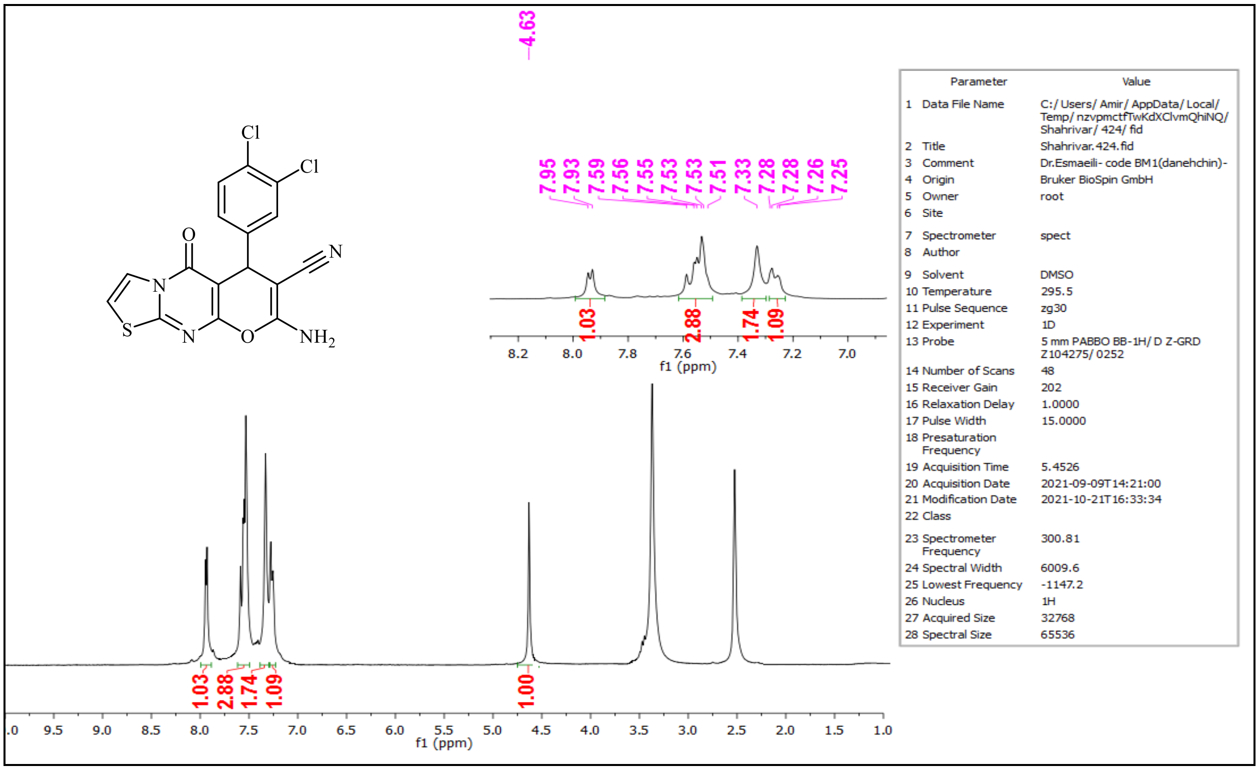


$^{1}$H NMR spectra of compound **4c**

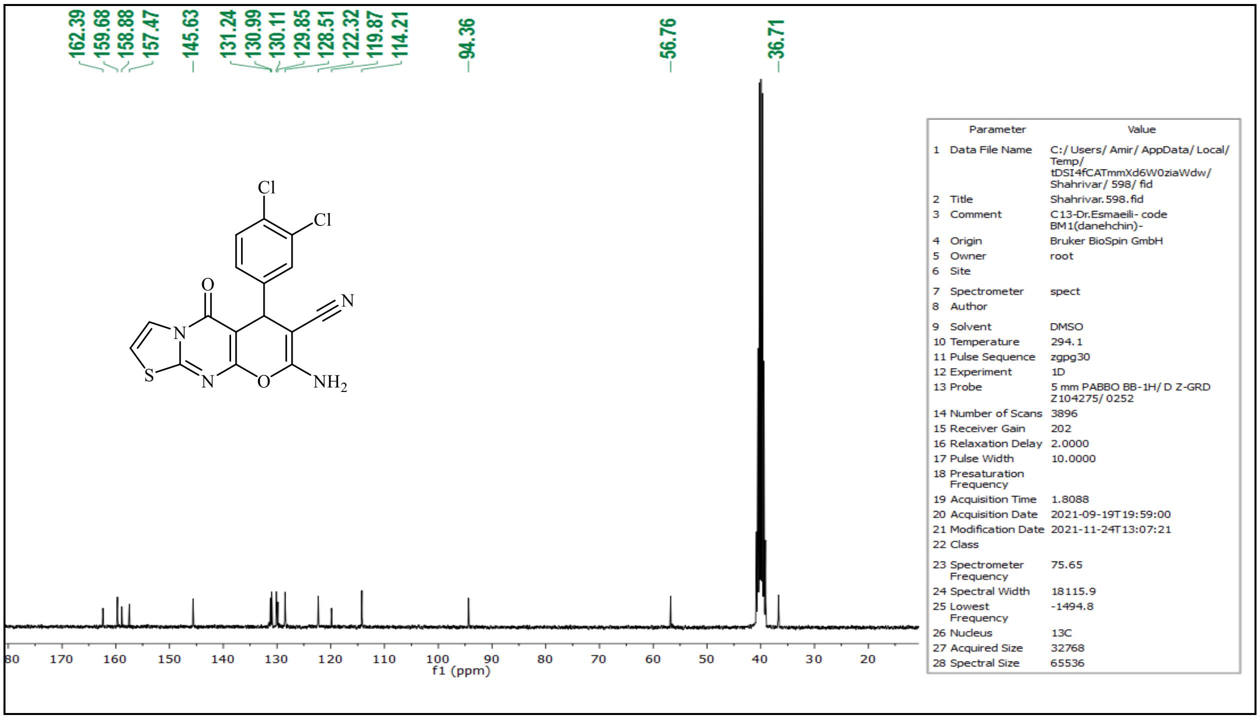


$^{13}$C NMR spectra of compound **4c**

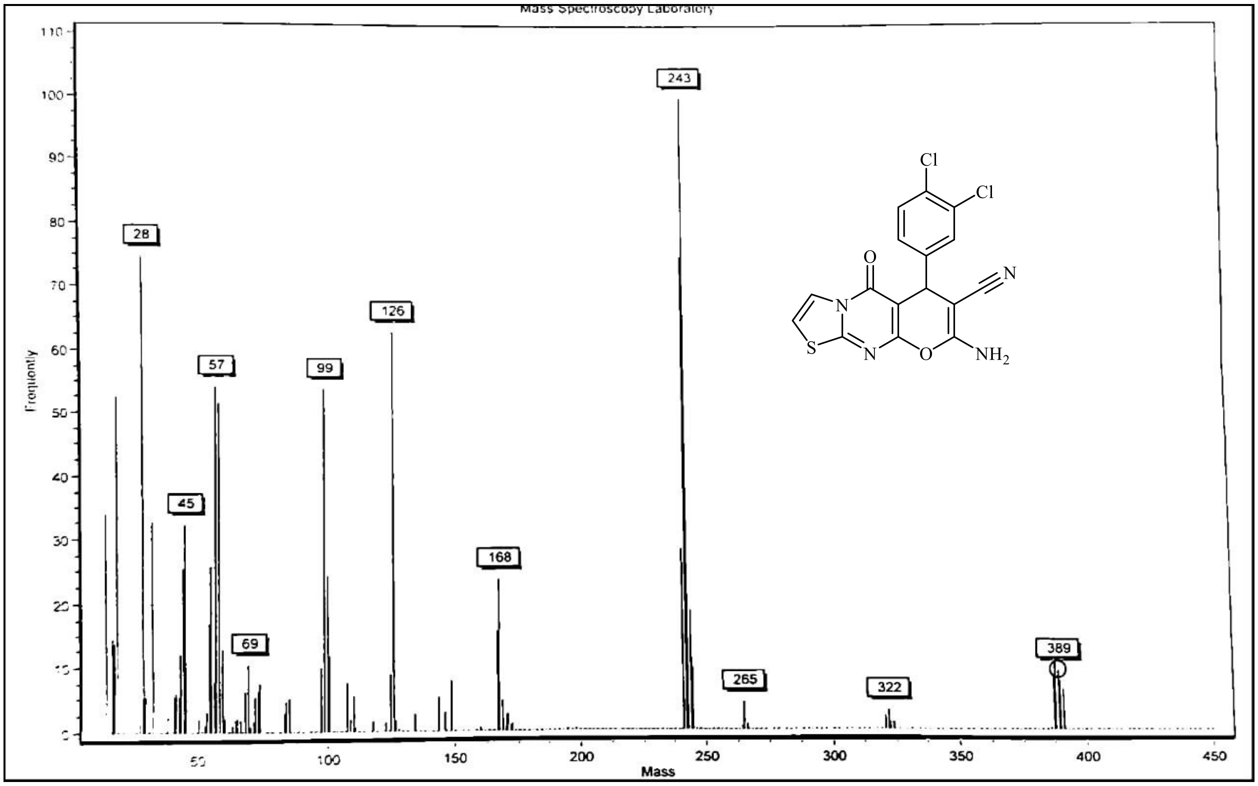


Mass spectra of compound **4c**

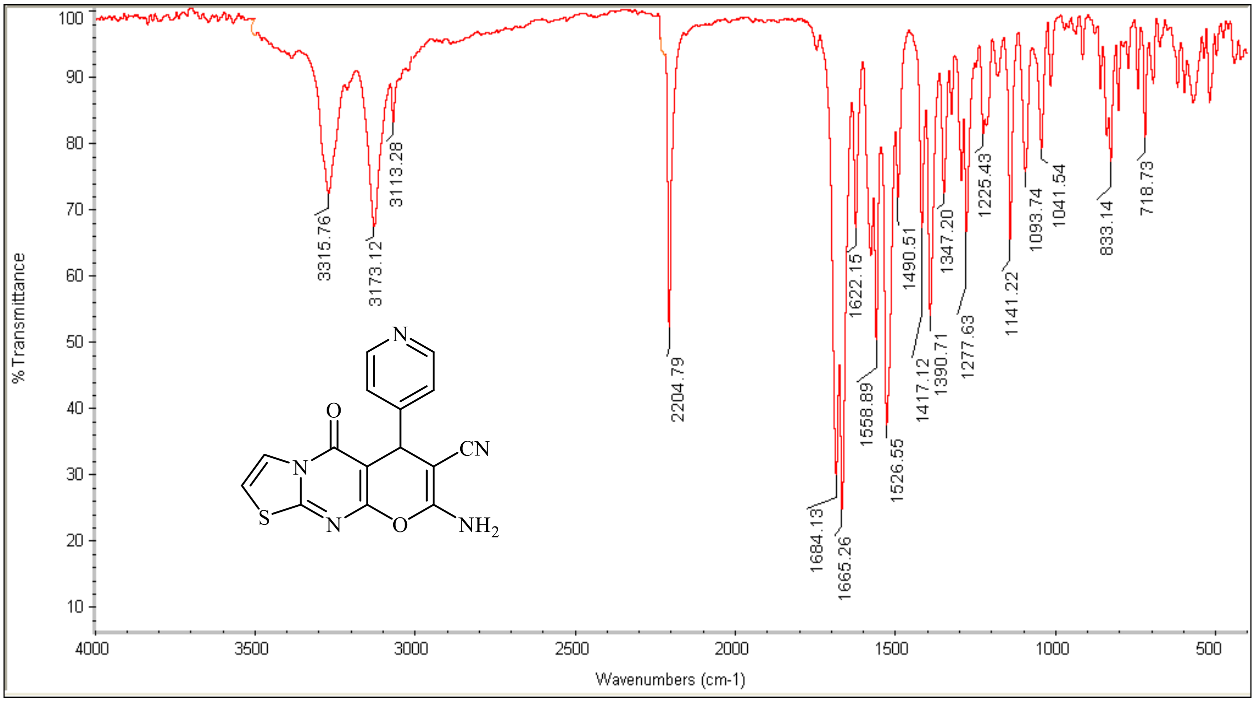


IR spectra of compound **4d**

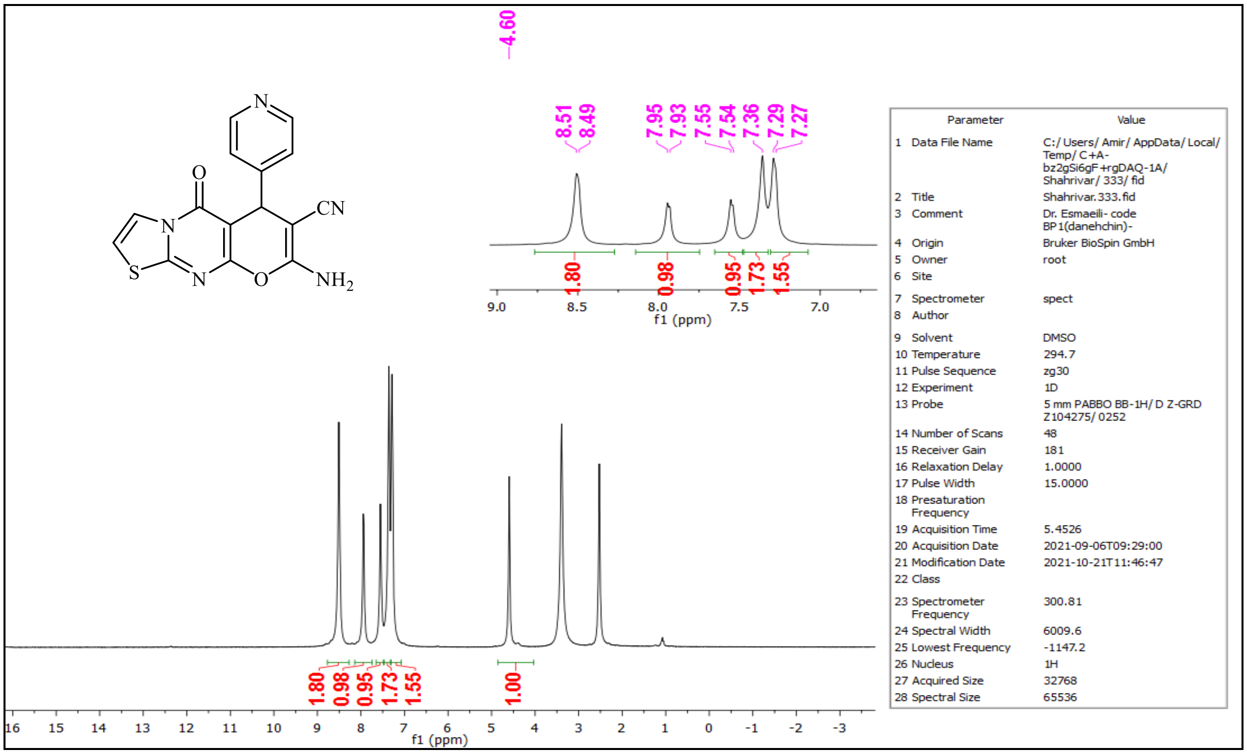


$^{1}$H NMR spectra of compound **4d**

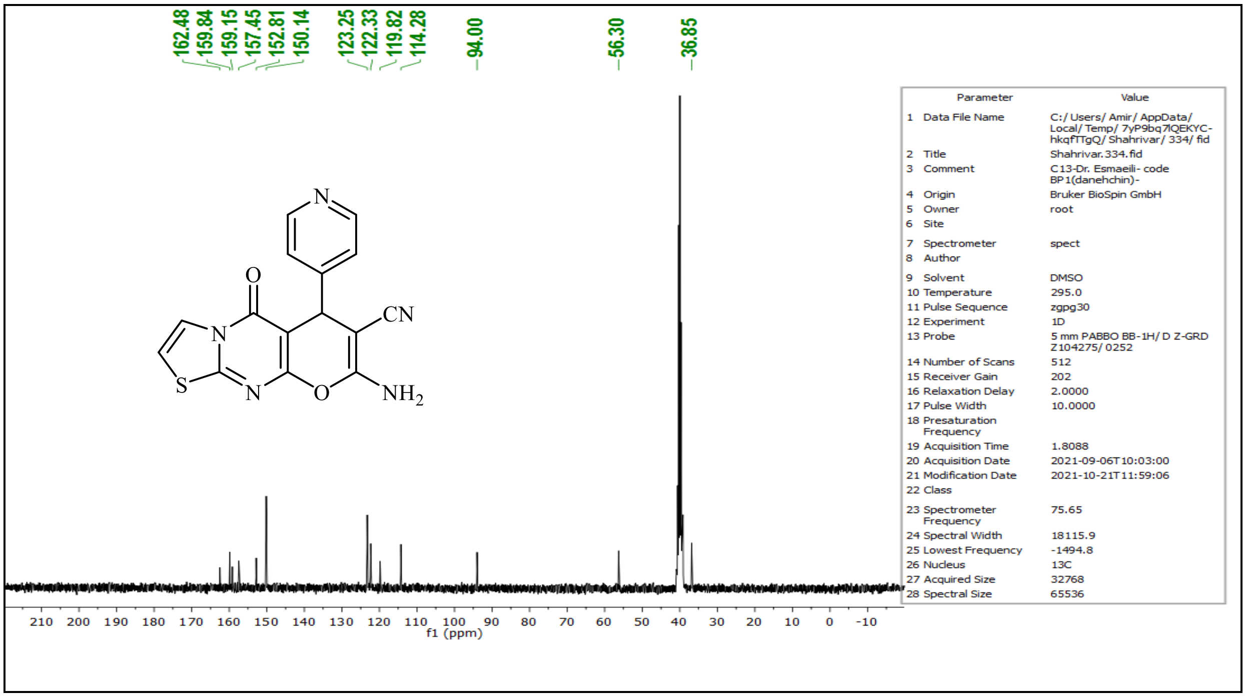


$^{13}$C NMR spectra of compound **4d**

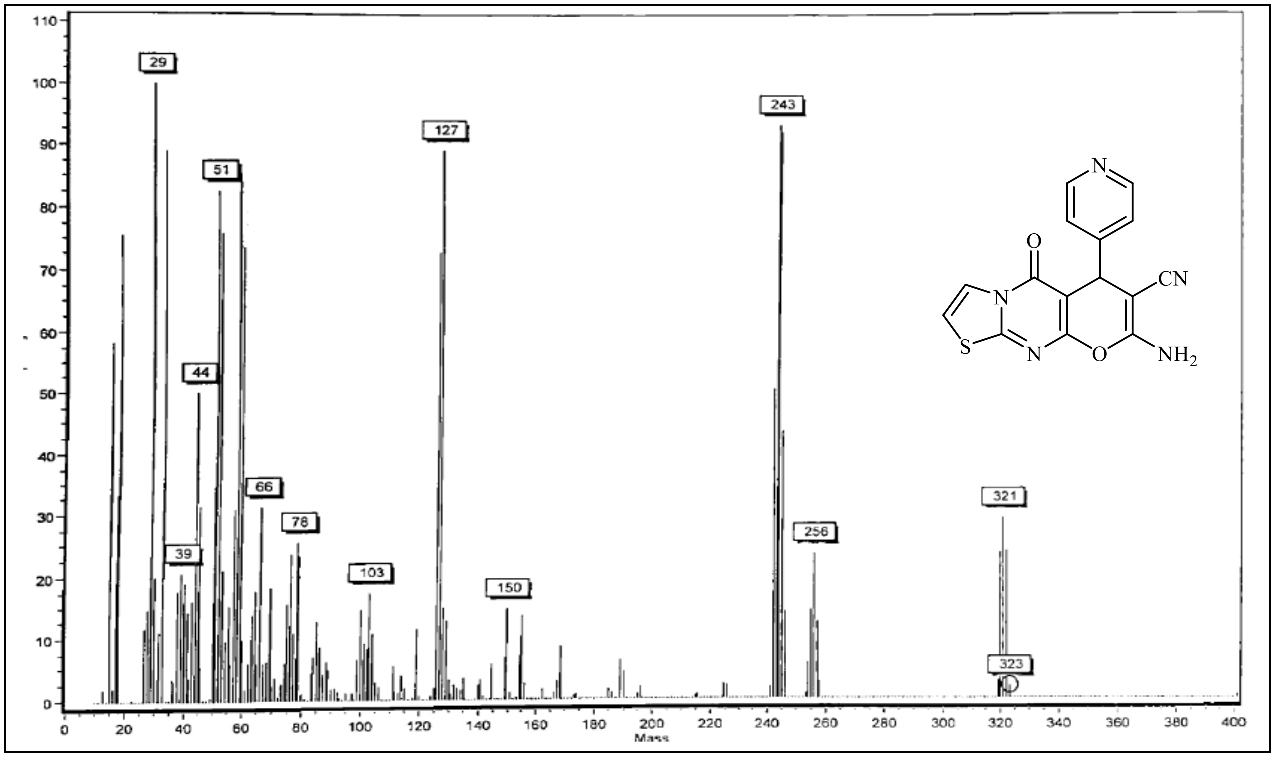


Mass spectra of compound **4d**

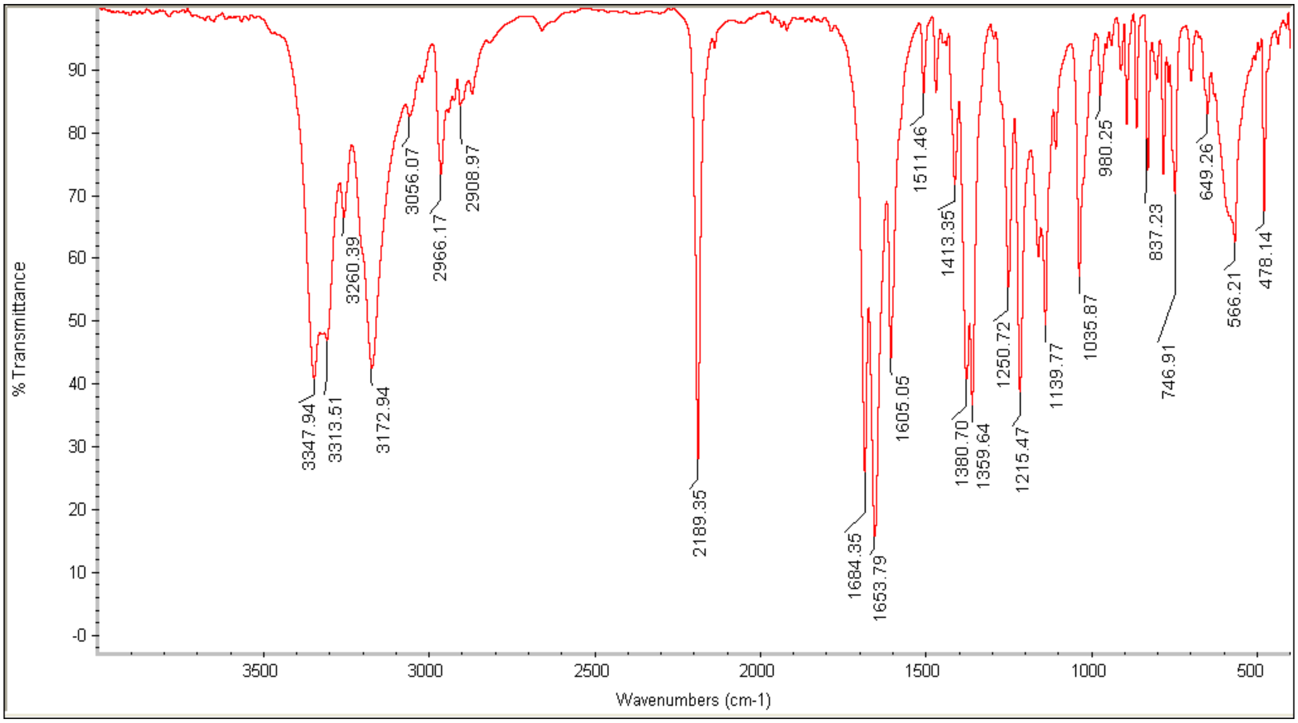


IR spectra of compound **6b**

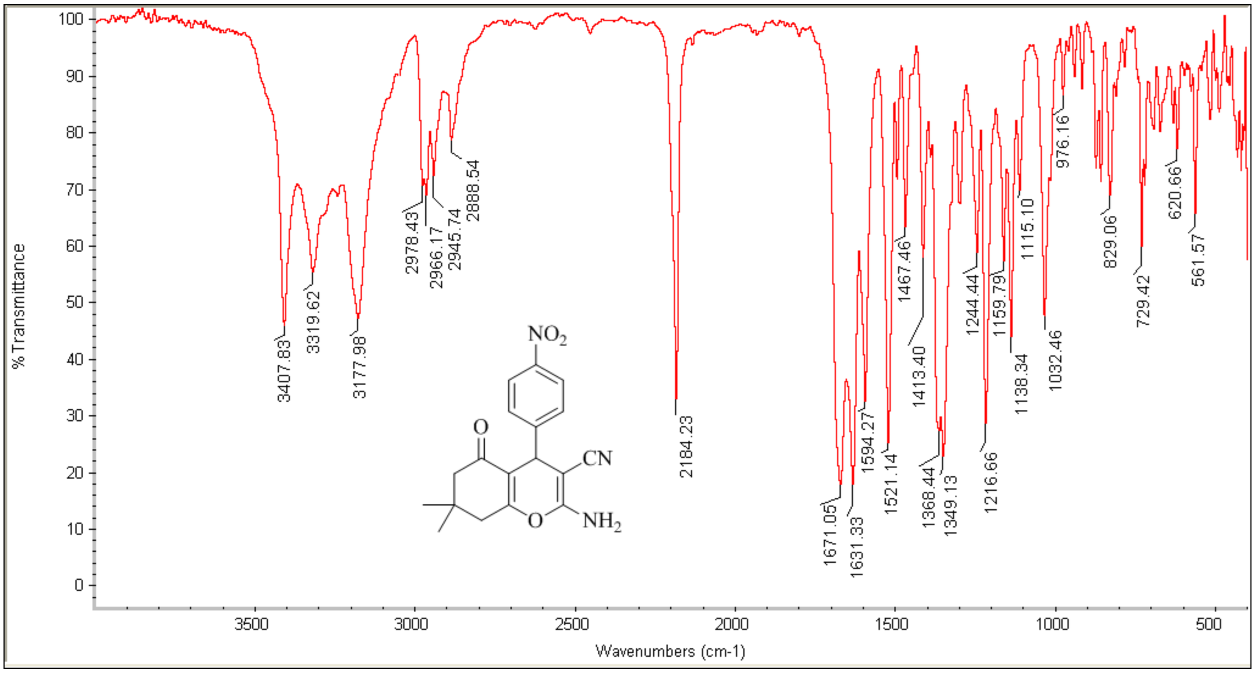


IR spectra of compound **6d**

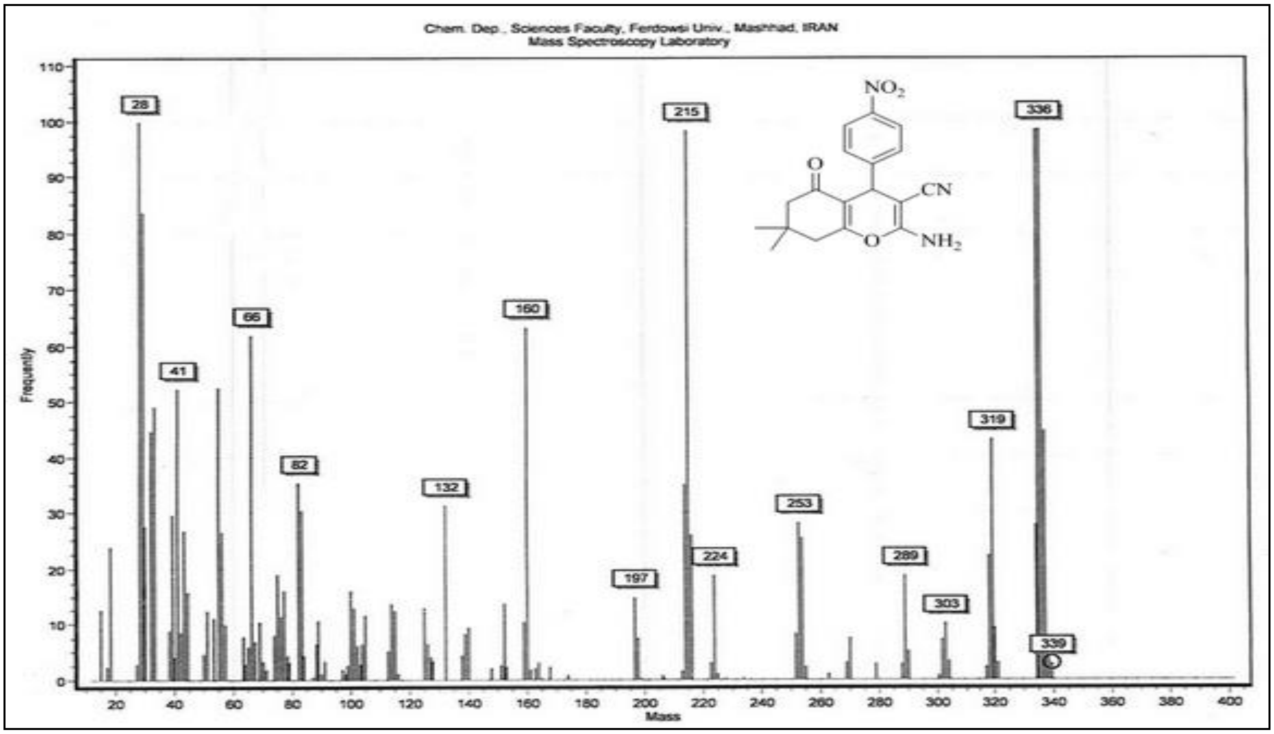


Mass spectra of compound **6d**

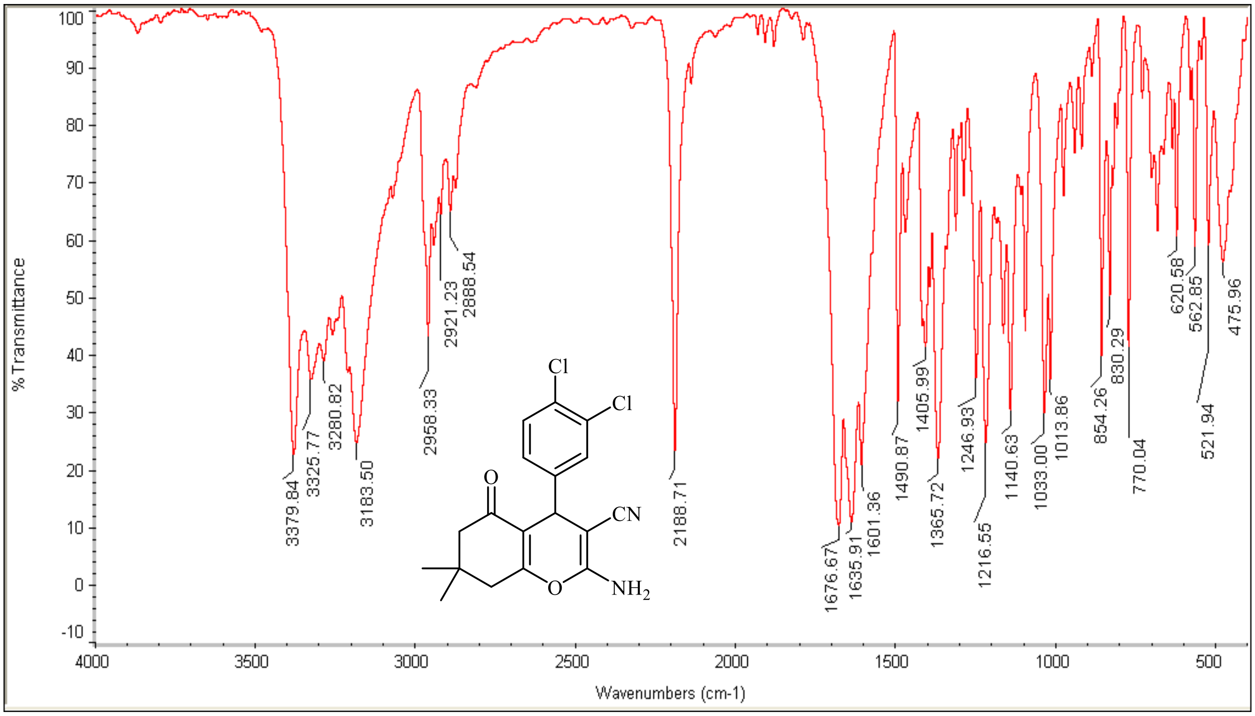


IR spectra of compound **6e**

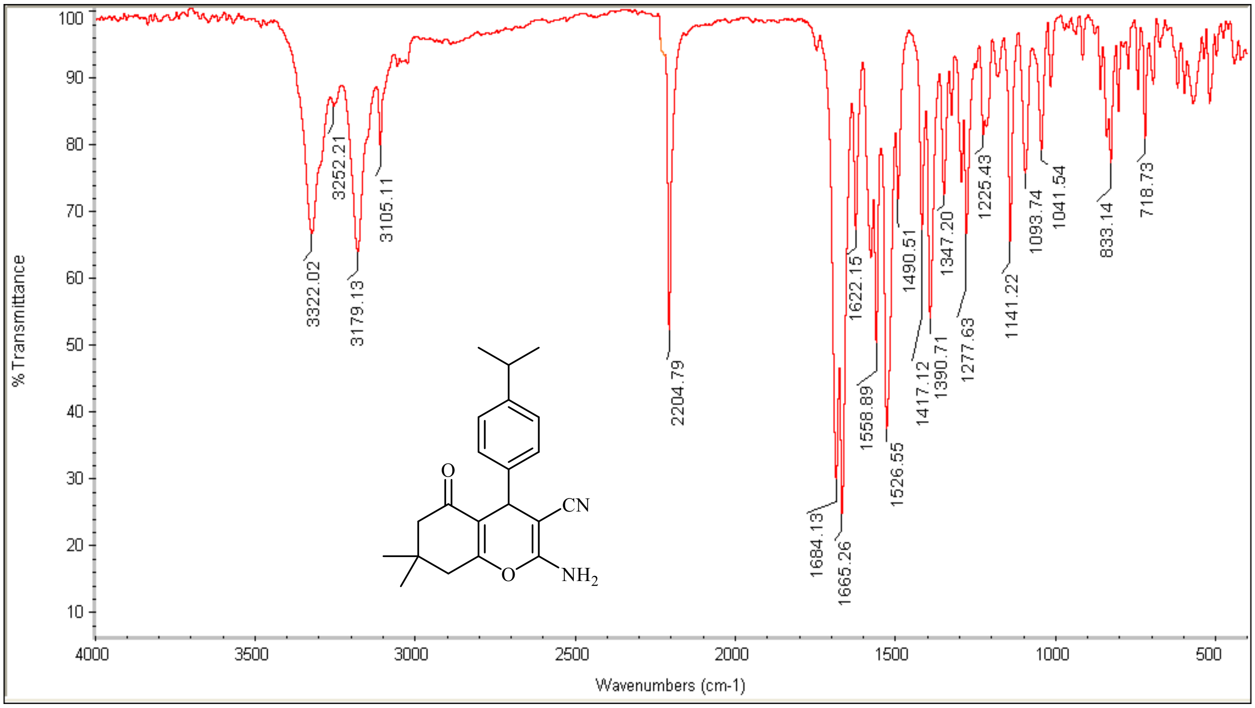


IR spectra of compound **6g**

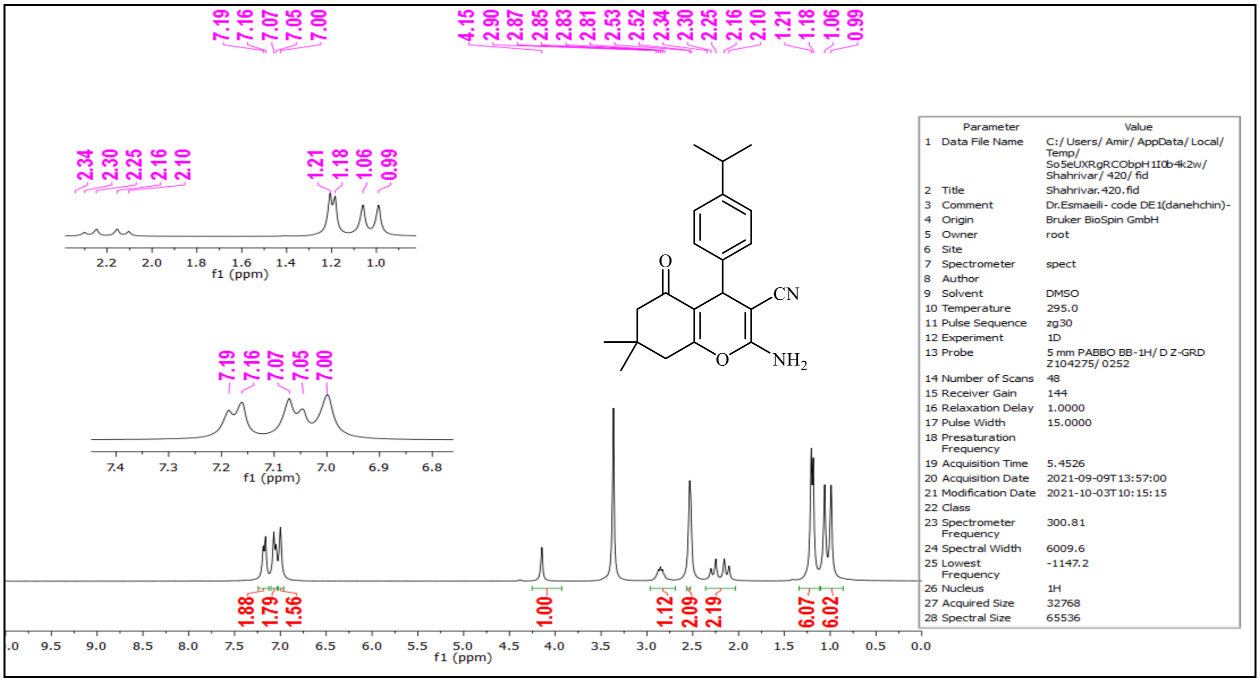


$^{1}$H NMR spectra of compound **6g**

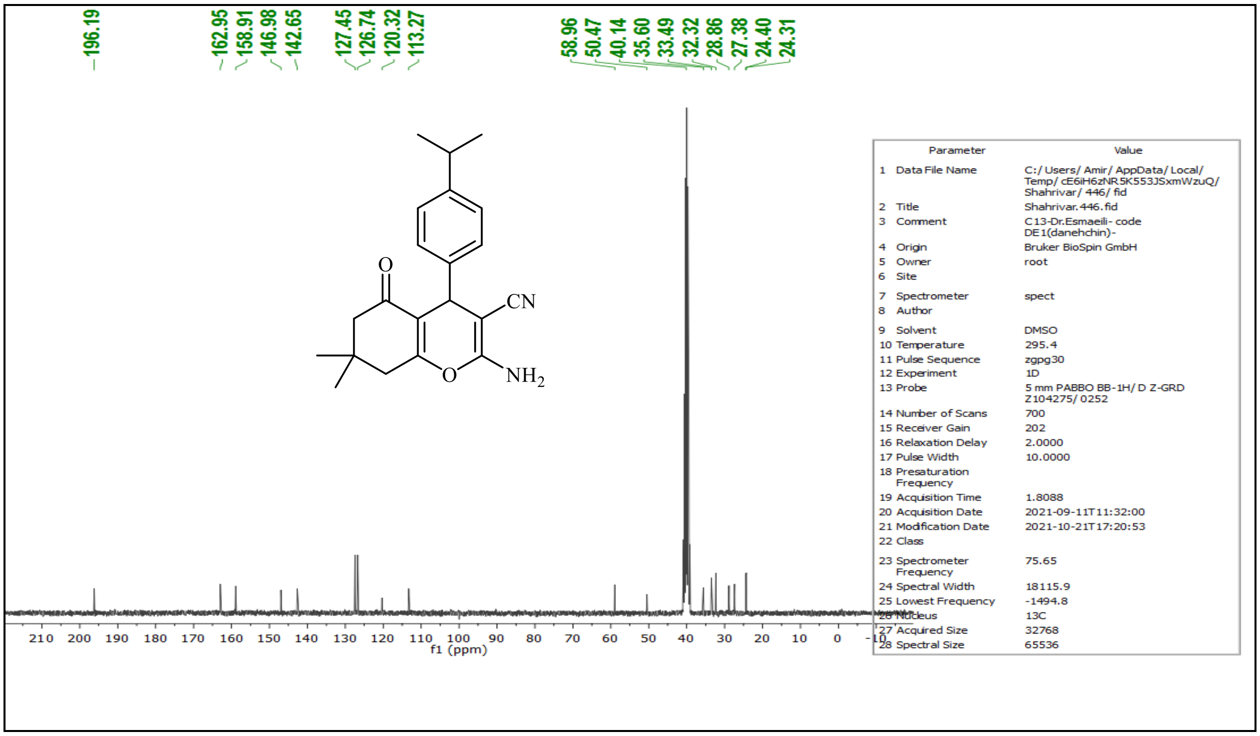

$^{13}$C NMR spectra of compound **6g**

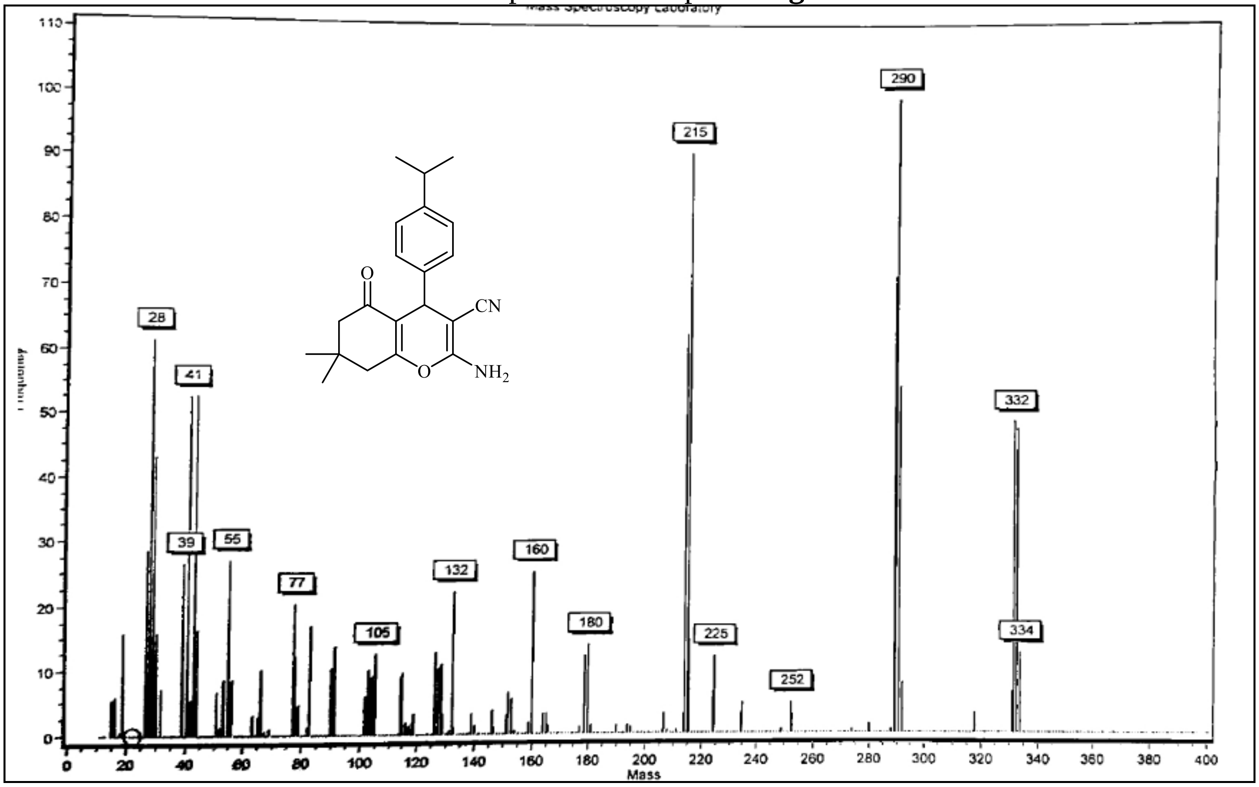

Mass spectra of compound **6g**

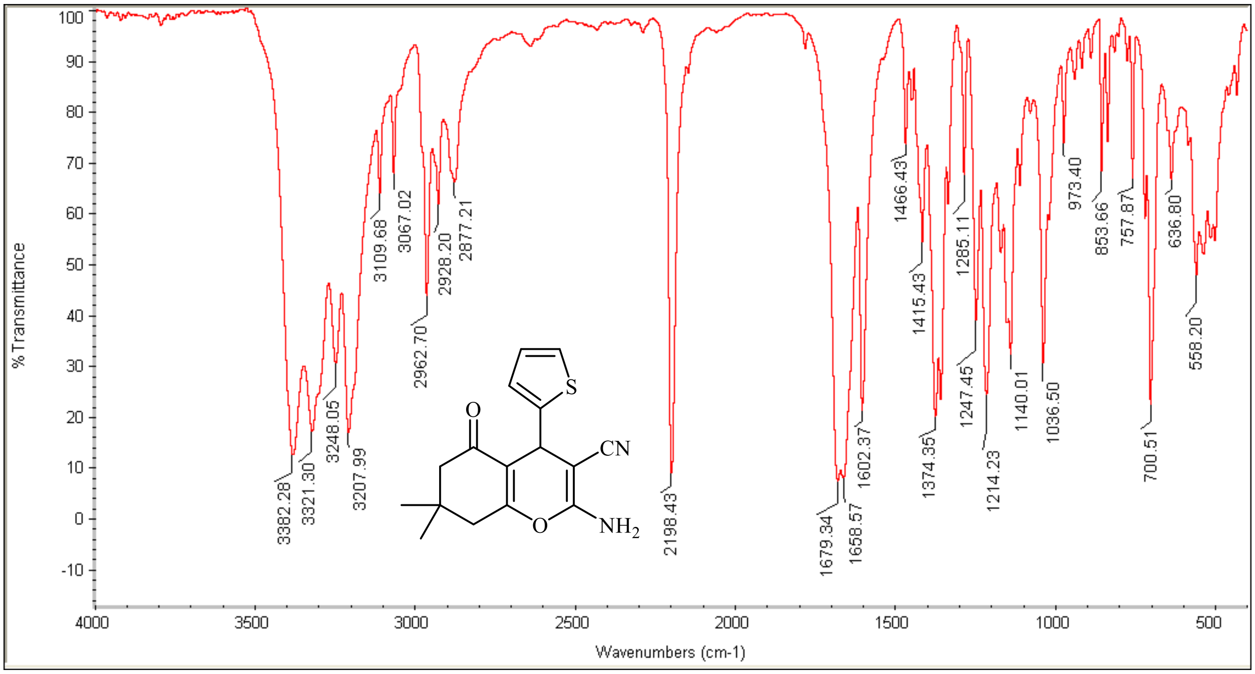


IR spectra of compound **6j**

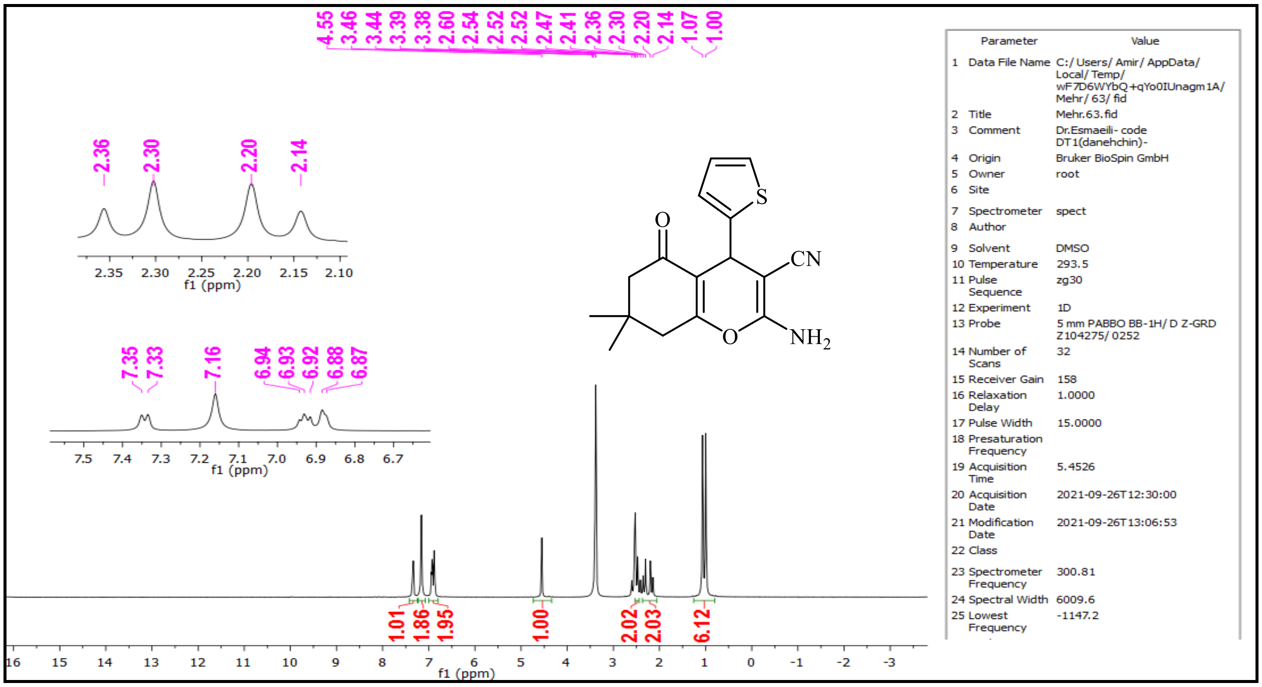


$^{1}$H NMR spectra of compound **6j**

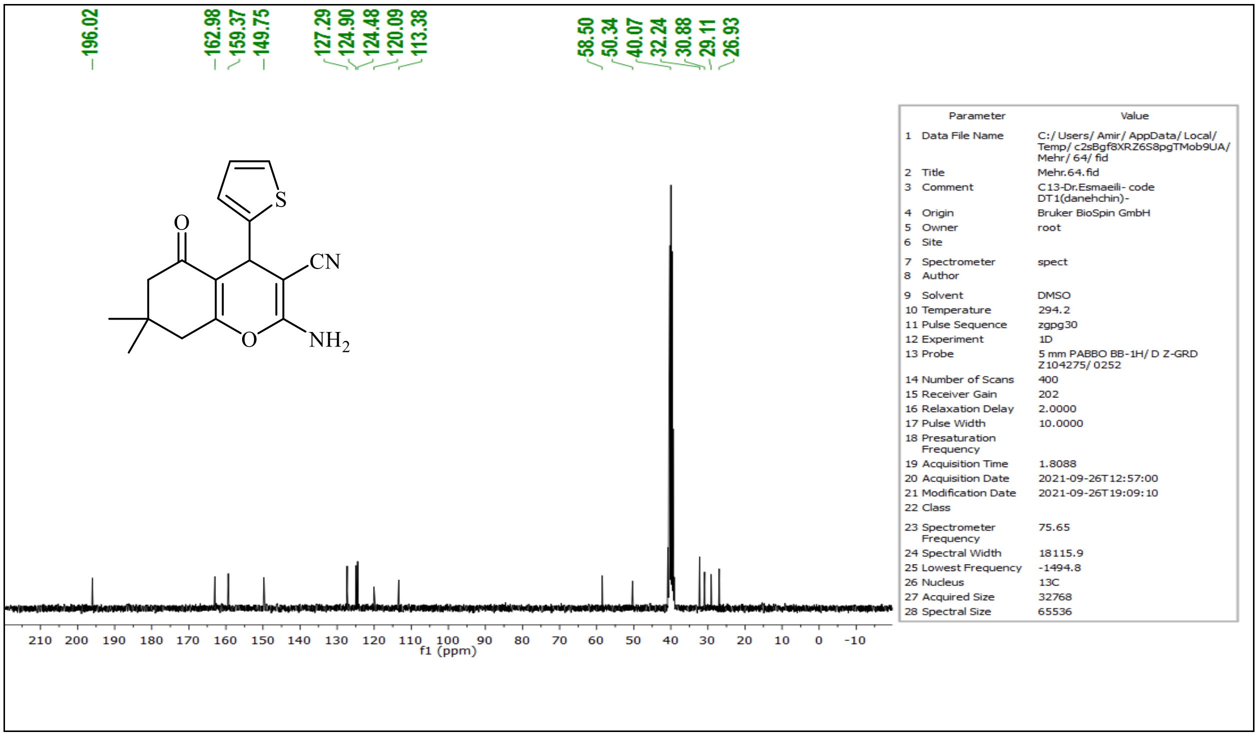


$^{13}$C NMR spectra of compound **6j**

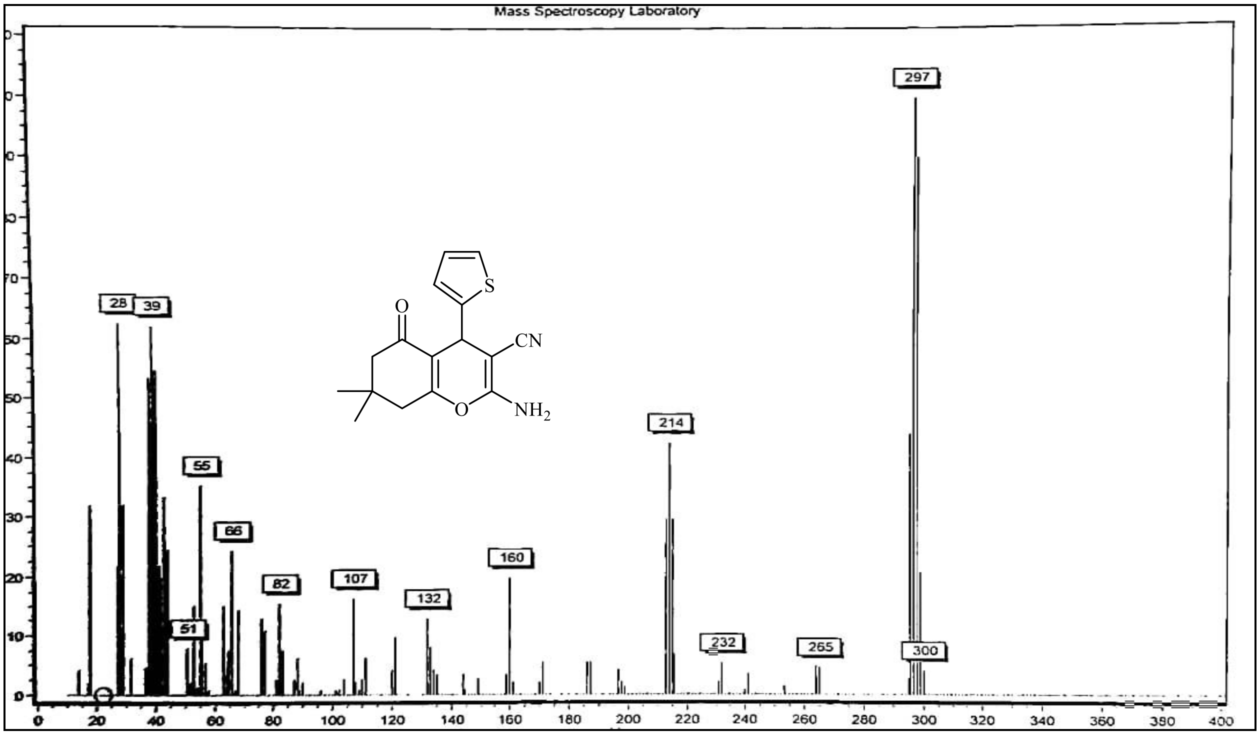


Mass spectra of compound **6j**

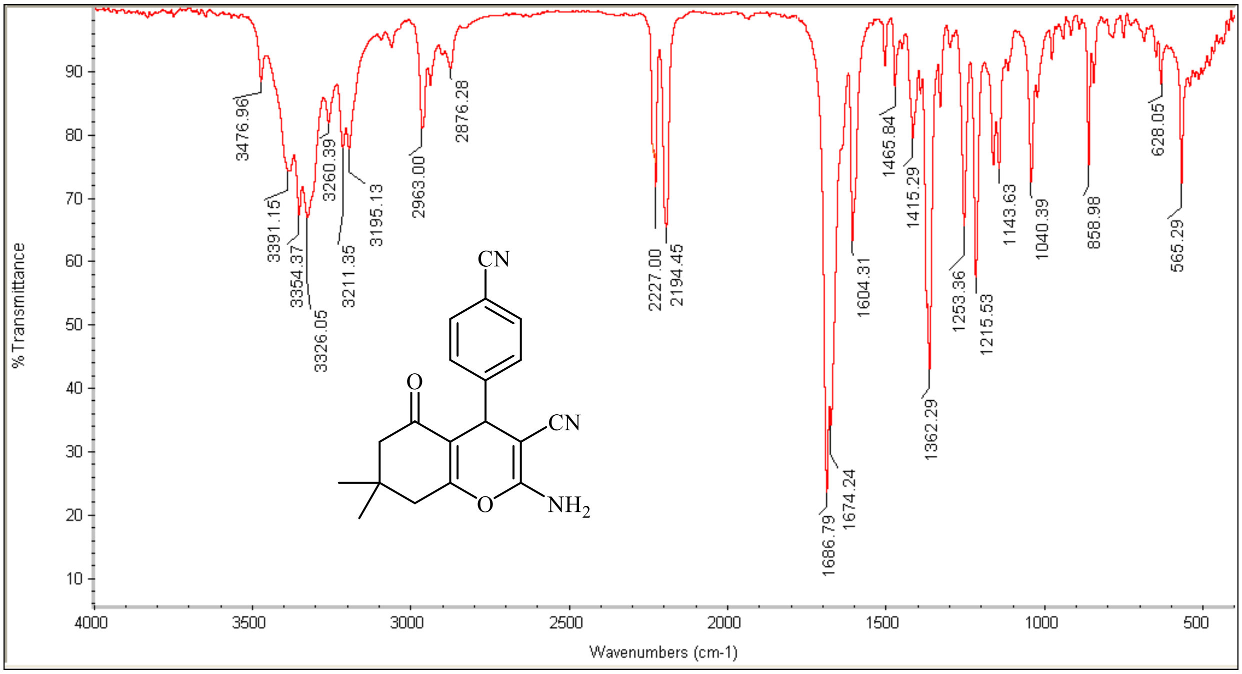


IR spectra of compound **6h**

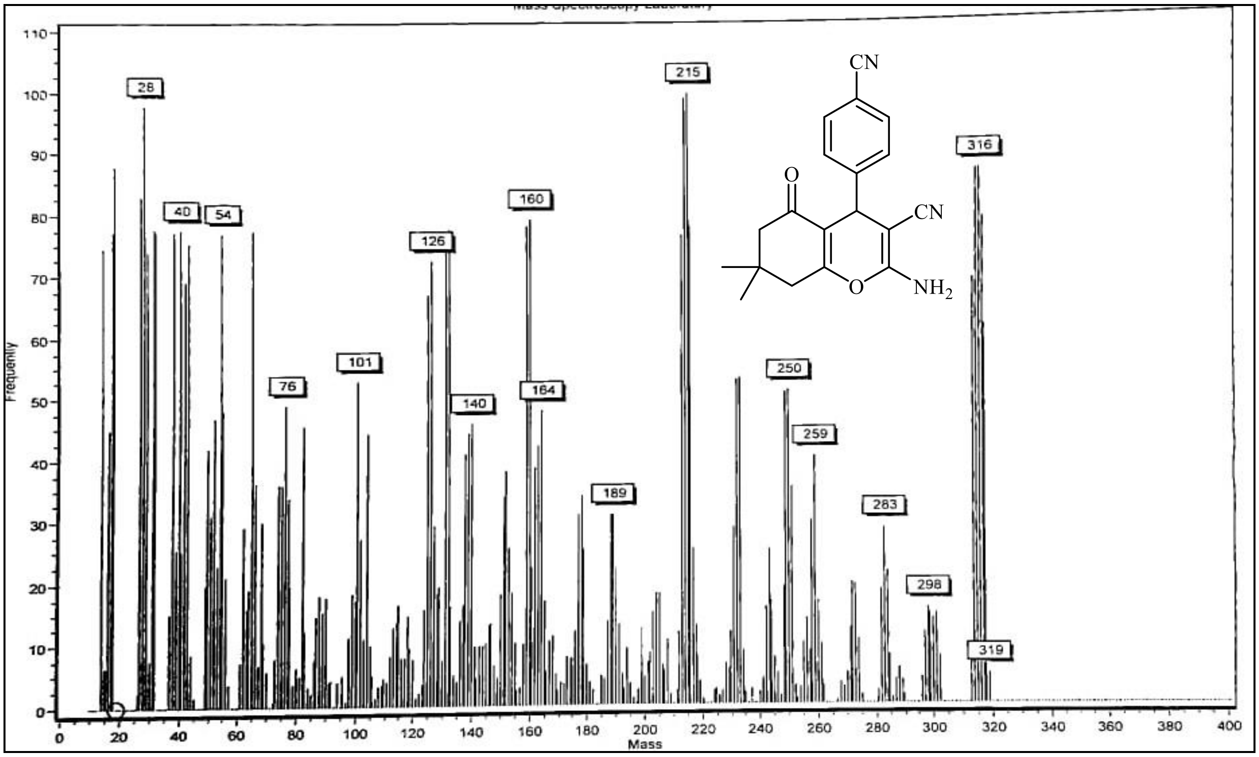


Mass spectra of compound **6h**

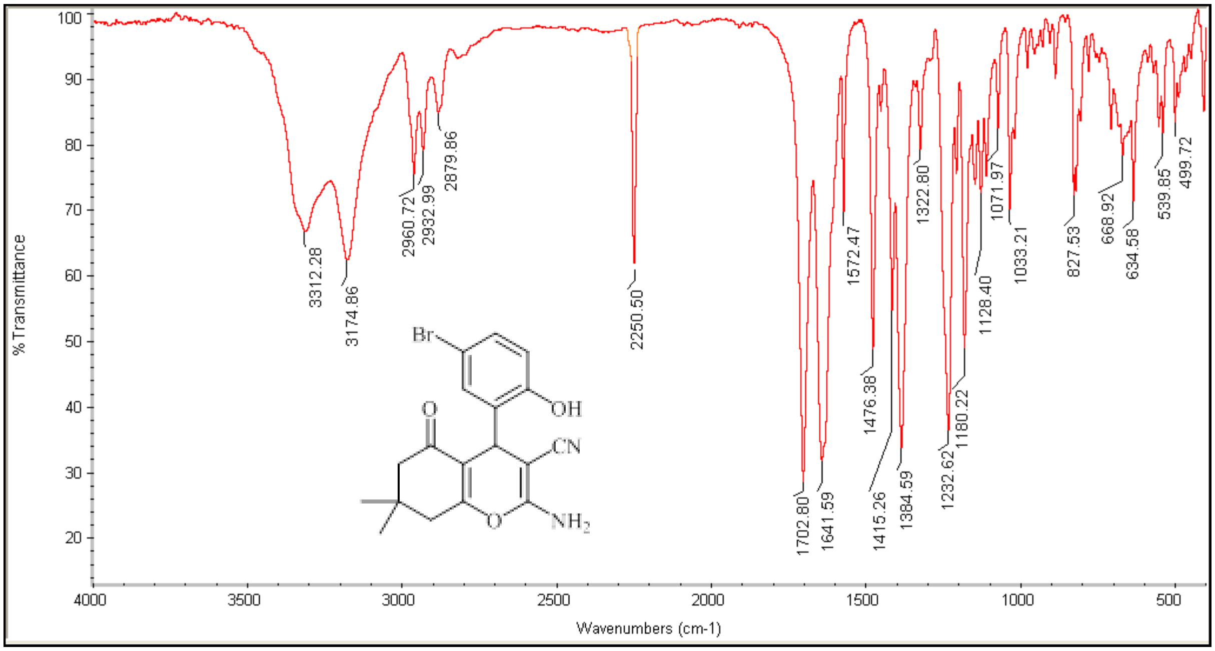


IR spectra of compound **6k**
